# Alkali Metal Complexes of a Phosphine‐Functionalized Cyclooctatetraene

**DOI:** 10.1002/chem.202502313

**Published:** 2025-08-11

**Authors:** Mohd Iqbal, Vanitha R. Naina, Xiaofei Sun, Shubham, Peter W. Roesky

**Affiliations:** ^1^ Institute of Inorganic Chemistry Karlsruhe Institute of Technology (KIT) Kaiserstr 12 76131 Karlsruhe Germany; ^2^ Institute for Nanotechnology Karlsruhe Institute of Technology (KIT) Kaiserstr 12 76131 Karlsruhe Germany

**Keywords:** alkali metals, cyclooctatetraene, inverse sandwich, phosphine, photoluminescent

## Abstract

The distinctive electronic properties and wide range of applications of cyclooctatetraene (COT) sandwich complexes make exploring this class of compounds essential. The new phosphine‐functionalized proligand 1,4−bis(dimethylsilylmethyl)diphenylphosphinecycloocta−2,5,7−triene, {C_8_H_8_−1,4−(Me_2_SiCH_2_PPh_2_)_2_}, was synthesized by reaction of K_2_COT with Me_2_Si(Cl)CH_2_PPh_2_ first. Subsequent deprotonation with various alkali metal bases led to a series of alkali metal complexes [M_2_{C_8_H_6_−1,4−(Me_2_SiCH_2_PPh_2_)_2_}] (M = Na, K, Rb, Cs). Notably, the crystallization solvent and the alkali metals significantly influenced the polymeric architecture and led to structural diversity of all complexes. For instance, the two phosphine arms either coordinate both to one metal atom, while the other metal ion is uncoordinated, or, in other structures, each metal atom is equally coordinated by just one phosphine function. Additionally, the coordination behavior of the pendant phosphine groups determines the emissive nature of these complexes. In all cases, polymeric structures are formed, in which intermolecular interactions between the COT ring and a metal atom are observed. Additionally, Rb forms a benzene‐bridged eight‐membered polymeric structure [{Rb_2_(C_8_H_6_−1,4−(Me_2_SiCH_2_PPh_2_)_2_) (C_6_H_6_)_2_}_8_]_∞_. Additionally, the lighter alkali metals in [M_2_{C_8_H_6_−1,4−(Me_2_SiCH_2_PPh_2_)_2_}] can be exchanged by the heavier congeners by using the corresponding *tert*−butoxides.

## Graphical abstract

A novel phosphine−functionalized COT ligand and its inverse sandwich alkali metal complexes (Na, K, Rb, and Cs) were synthesized and structurally characterized. The polymeric architectures vary with the metal ion and crystallization solvent, with an eight‐membered ring obtained for Rb in benzene. UV−Vis and photophysical studies reveal luminescent properties.

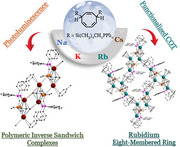

## 1 Introduction

**Figure 1:**
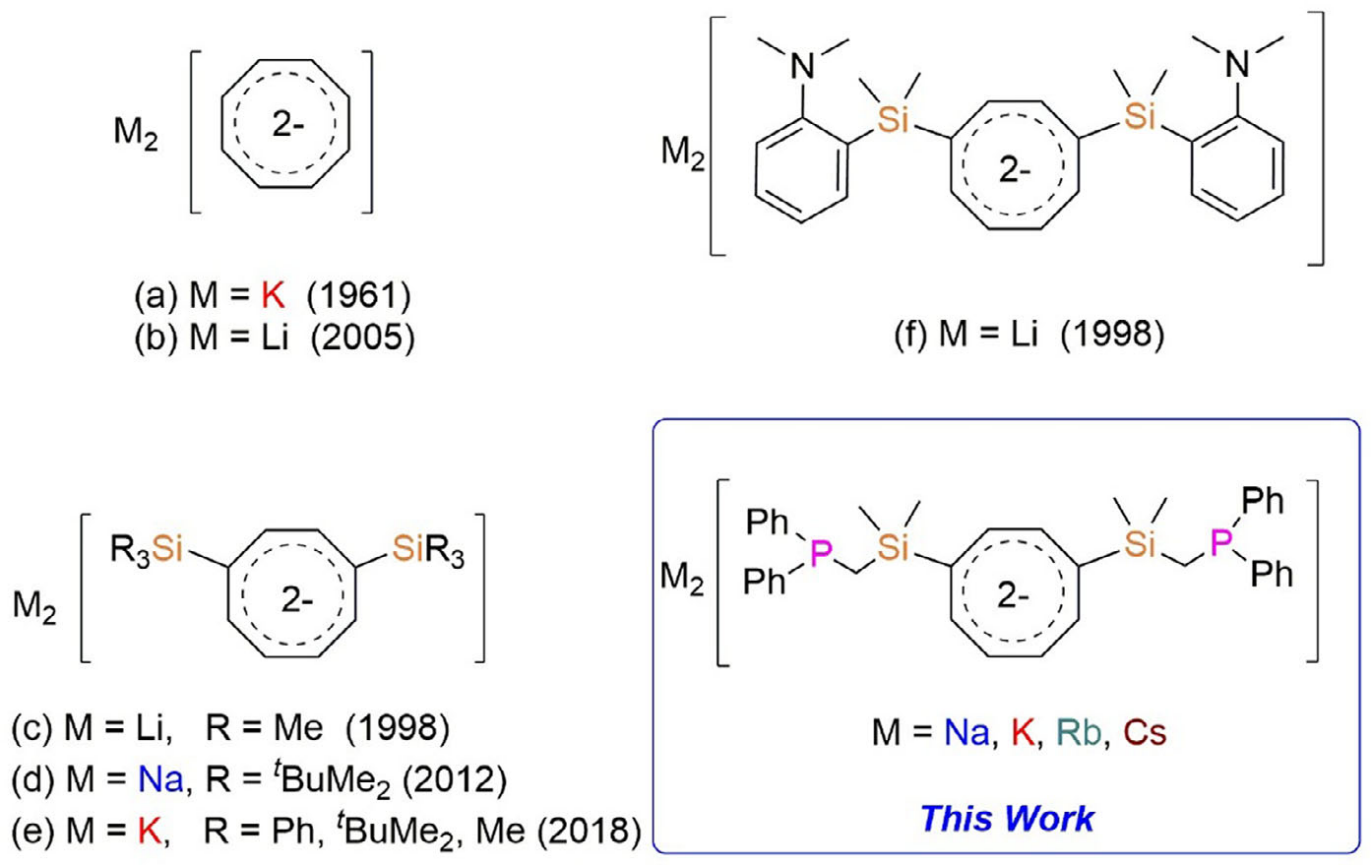
(a) and (b): Reported examples of structurally characterized cyclooctatetraenide; (c), (d) and (e): silyl‐substituted cyclooctatetraenide; (f), (d): N‐donor functionalized substituted cyclooctatetraenide (in solution); and this work.

Cyclooctatetraene (COT) has played a significant role in advancing organometallic chemistry since the seminal discovery of uranocene by Streitwieser and Müller‐Westerhoff in 1968.^[^ ^1^ ^]^ Initially, COT was characterized as an eight *π*‐electron system and identified as a nonplanar, nonaromatic molecule due to its tub‐shaped geometry. However, upon reduction by two *π*‐electrons, the cyclooctatetraene dianion (COT^2^−) adopts a planar and aromatic structure.^[^ ^2^, ^3^, ^4^, ^5^ ^]^ The first reduction of COT to form the aromatic cyclooctatetraenide dianion, a flat 10 *π*‐electron system, was reported by Katz in 1960 using potassium as a reducing agent.^[^ ^6^ ^]^ Since then, alkali metal salts of COT, particularly those with lithium and potassium, have emerged as versatile reagents in the synthesis of diverse complexes involving transition metals, lanthanides, and actinides.^[^ ^6^, ^7^, ^8^, ^9^, ^10^, ^11^, ^12^, ^13^ ^]^ The first structurally confirmed aromatic dianion K_2_C_8_H_8_·THF, was reported by Fritz and Keller in 1961 (a in Figure 1).^[^ ^14^ ^]^ Later, Gausing and Wilke disclosed the first synthesis of di‐lithium COT using three equivalents of *n*‐butyllithium (*n*‐BuLi) in the presence of tetramethyl ethylenediamine (TMEDA) (b in Figure 1).^[^ ^15^, ^16^ ^]^ However, the unsubstituted COT poses many challenges in organometallic chemistry, including a lack of solubility and stability, particularly for heavier metals.^[^ ^17^, ^18^ ^]^

Over the past three decades, there has been increasing interest in silyl‐substituted COT ligands, such as C_8_H_6_−1,4−(Me_3_Si)_2_, C_8_H_6_−1,4−(Me_2_ *^t^* BuSi)_2_, and C_8_H_5_− 1,3,6−(Me_3_Si)_3_.^[^ ^19^ ^]^ As disclosed by the F. G. N. Cloke group, the silyl groups can be bound in high yield and selectivity to the eight‐membered ring.^[^ ^20^ ^]^ Notably, the COT ring often demands bulky substitutions to stabilize the heavier metal center in complexes. Recently, a new dimension in steric bulk was introduced to this chemistry by the introduction of the “super bulky” ligands {C_8_H_6_−1,4−(SiR_3_)_2_} (R = *^i^* Pr, Ph).^[^ ^12^, ^21^, ^22^, ^23^, ^24^, ^25^ ^]^ The substitution plays a critical role in lanthanide and actinide complex architectures, particularly in forming sandwich and multidecker sandwich complexes.^[^ ^16^, ^26^, ^27^, ^28^, ^29^ ^]^ Furthermore, these silyl substitutions significantly alter the solubility, reactivity, and electronic properties of the resulting metal complexes.^[^ ^22^, ^23^, ^24^, ^26^, ^27^, ^28^, ^29^, ^30^, ^31^, ^32^, ^33^, ^34^, ^35^, ^36^, ^37^, ^38^, ^39^ ^]^ Over the last few years, our group has also been working on unsubstituted COT and silyl‐substituted COT‐based ligands in lanthanide chemistry.^[^ ^23^, ^40^, ^41^, ^42^, ^43^, ^44^, ^45^ ^]^

The alkali metal salts of COT‐based ligands serve as essential precursors in lanthanide and actinide chemistry.^[^ ^19^, ^20^, ^26^, ^27^, ^29^, ^31^, ^32^, ^34^, ^46^, ^47^, ^48^, ^49^ ^]^ However, only a limited number of alkali metal complexes featuring silyl‐substituted COT ligands have been successfully isolated and fully characterized (c, d and, e in Figure 1).^[^ ^14^, ^15^, ^16^, ^38^, ^50^, ^51^ ^]^ The first crystallographic characterization of a substituted COT complex, specifically the di−lithium salt, [Li_2_{C_8_H_6_−1,4−(Si(*^t^* BuMe_2_)_2_}] was reported in 1998 by F.T. Edelmann and coworkers.^[^ ^50^ ^]^ The synthetic methodology followed the reaction of 1,5−cyclooctadiene (1,5‐COD) with three equivalents of *n*‐BuLi in the presence of TMEDA, yielding the di‐lithium COT complex.^[^ ^50^ ^]^ The first crystal structure of a sodium derivative, [Na_2_{C_8_H_6_−1,4−(SiMe_3_)_2_}(*μ*−THF)_2_], was elucidated in 2012 by the M. Murugesu group.^[^ ^51^ ^]^ Later in 2017, Lorenz *et al.* synthesized and structurally characterized a series of potassium COT complexes, including [K_2_{C_8_H_6_‐1,4‐(SiMe_3_)_2_}], [K_2_{C_8_H_6_‐1,4‐(Si(*^t^* BuMe_2_)_2_}], and [K_2_{C_8_H_6_‐1,4‐(SiPh_3_)_2_}].^[^ ^38^ ^]^ Recently, in 2022, dibenzo COT was comprehensively investigated across the alkali metal series by the M. A. Petrukhin group.^[^ ^52^ ^]^ While Li, Na, and K with silyl‐substituted COT derivatives are well established, the corresponding Rb and Cs compounds remain unknown.

While donor‐functionalized cyclopentadienyl ligands in metal complexes, for example, Josiphos^[^ ^53^, ^54^ ^]^ or 1,1′‐bis(diphenylphosphino)ferrocene (dppf),^[^ ^55^ ^]^ are established and commercially available, only a few donor‐substituted COT derivatives are known, for example, C_8_H_7_NMe_2_^[^ ^56^ ^]^ and C_8_H_7_PR_2_ (R = Et, *^t^* Bu, Ph).^[^ ^56^ ^]^ They were mainly investigated in uranium chemistry in the 1970s. While the amino‐functionalized uranocenes such as [U(C_8_H_7_NMe_2_)_2_] and [U(C_8_H_7_CH_2_NMe_2_)_2_] were reported,^[^ ^56^ ^]^ for C_8_H_7_PR_2_ (R = Et, *^t^* Bu, Ph), no pure uranocene derivatives were obtained.^[^ ^56^ ^]^ In contrast, similar investigations of the lanthanides are scarce. To date, only a single example of a *N*‐donor functionalized di‐substituted COT ligand has been reported (f, in Figure 1).^[^ ^57^ ^]^ This COT ligand, which is functionalized by *o*‐dimethyl silyl‐*N,N*‐dimethylaniline was subsequently explored in lanthanide coordination chemistry.^[^ ^57^, ^58^ ^]^ Due to the rigid aniline scaffold, the *N*‐donor atom could be neither intramolecularly nor intermolecularly coordinated to any metal atom.

Since phosphine‐substituted cyclopentadienyl ligands are frequently used, we were challenged to synthesize a related COT ligand. Herein, we report the coordination chemistry of the 1,4−bis (dimethylsilylmethyl) diphenylphosphinecycloocta‐1,3,5,7‐tetraene dianion ({C_8_H_6_−1,4−(Me_2_SiCH_2_PPh_2_)_2_}^2−^) in alkali metal chemistry. This approach led to the formation of a series of inverse sandwich polymeric derivatives. These complexes were further studied for their photophysical properties.

## 2 Results and Discussion

### 2.1 Synthesis and Characterization

Due to the pioneering work of the F. G. N. Cloke group, it is known that R_3_Si (R = Alkyl, Aryl) groups can be selectively coordinated in a 1,4−position to the COT ring.^[^ ^20^ ^]^ The traditional methods for preparing these silyl‐substituted COT ligands typically involve the reaction of K_2_COT or Li_2_COT precursors with R_3_SiCl.^[^ ^20^, ^24^, ^59^ ^]^ Direct substitution of functional groups onto COT is challenging due to its high basicity, which often leads to elimination reactions.^[^ ^57^, ^58^ ^]^ To overcome this issue, our group previously employed a silyl‐bridging to introduce the *o*−dimethyl silyl−*N,N*−dimethylaniline function.^[^ ^57^ ^]^ Now, this same synthetic methodology was successfully extended to substitute diphenyl phosphine group onto the COT ring.

**Scheme 1:**
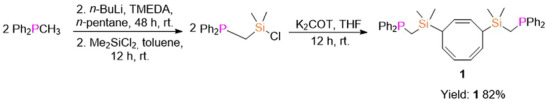
Synthesis of {C_8_H_8_−1,4−(Me_2_SiCH_2_PPh_2_)_2_} (**1**).

For the synthesis of 1,4−bis(dimethylsilylmethyl)diphenylphosphinecycloocta−2,5,7−triene, {C_8_H_8_−1,4−(Me_2_SiCH_2_PPh_2_)_2_} (1) (Scheme 1), we used Me_2_Si(Cl)CH_2_PPh_2_ as a precursor, which was prepared following a reported method starting from Ph_2_PMe.^[^ ^60^ ^]^ In the salt metathesis reaction, two equivalents of Me_2_Si(Cl)CH_2_PPh_2_ and one equivalent of K_2_COT in tetrahydrofuran (THF) were combined at −30 °C. Gradually warming to room temperature over 12 hours yielded the desired compound **1** as a brown−yellow oily liquid. The proligand **1** acquired thermal stability up to ∼150 °C but is moisture sensitive and decomposes upon prolonged storage at room temperature or −30 °C. To prevent degradation, the compound was used immediately in subsequent reactions. The composition of ligand **1** was established by ^1^H, ^13^C{^1^H}, ^31^P{^1^H}, and ^29^Si{^1^H} NMR spectroscopy. The substitution pattern of **1** was readily identified by ^1^H NMR spectroscopy (Figure S1), particularly from the signals corresponding to the eight‐membered ring, which were consistent with those previously reported for similar silyl‐substituted ligands.^[^ ^24^, ^38^, ^57^ ^]^ In the ^31^P{^1^H} NMR spectrum, the phosphorus resonance was observed at *δ* = −22.1 ppm (Figure S3), slightly downfield‐shifted compared to the Me_2_Si(Cl)CH_2_PPh_2_ precursor *δ* = −24.0 ppm,^[^ ^60^ ^]^ indicating successful substitution. Additionally, the ^29^Si{^1^H} NMR spectrum displayed a doublet signal at *δ* = 5.2 ppm (d, *J*_SiP_ = 15.2 Hz) (Figure S4).

**Scheme 2:**
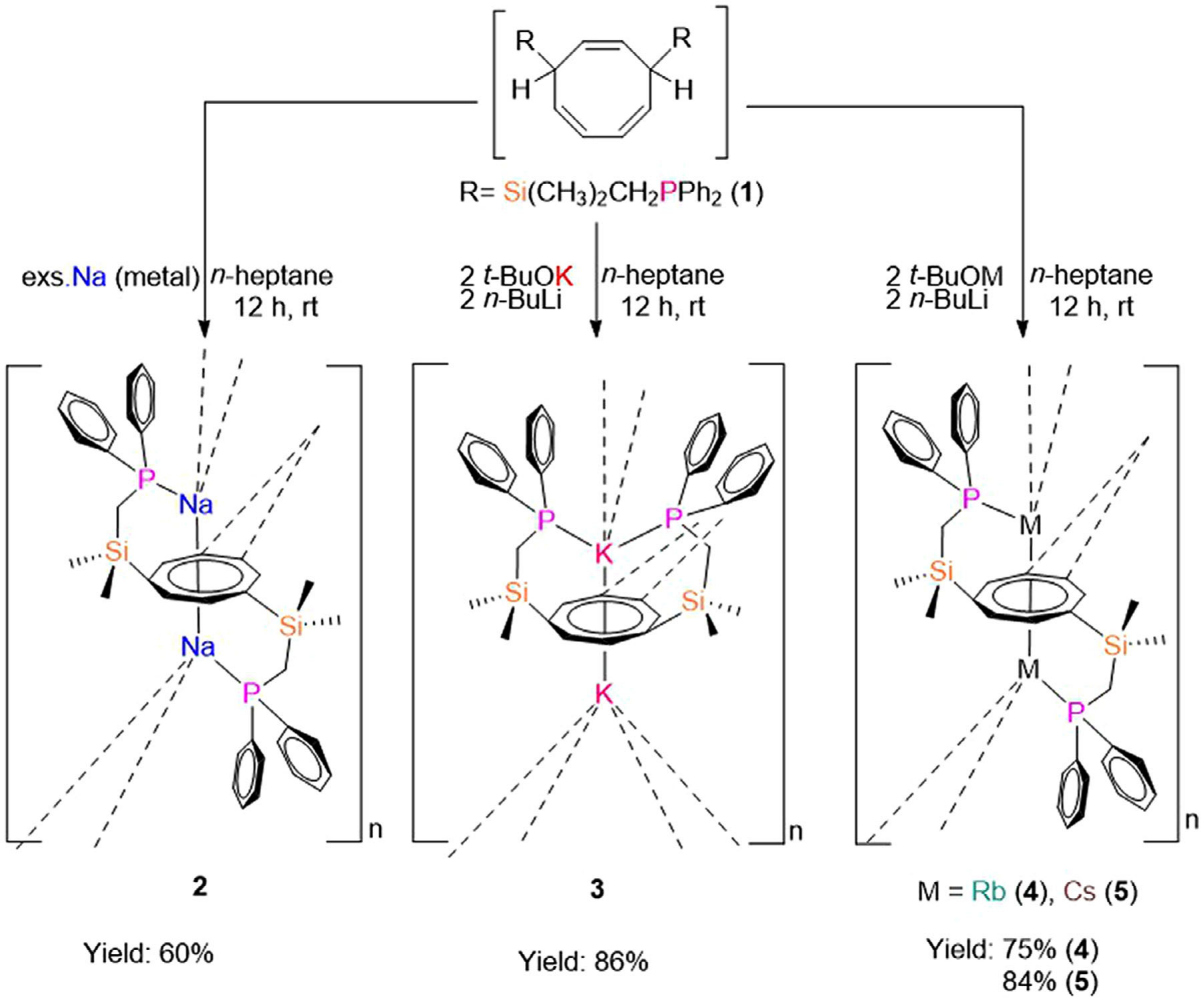
Synthesis of [M_2_{C_6_H_6_‐1,4−(Me_2_SiCH_2_PPh_2_}_2_] (M = Na (**2**), K (**3**), Rb (**4**), Cs (**5**)).

Subsequently, we utilized compound **1** in the synthesis of alkali metal inverse sandwich complexes, [M_2_{C_8_H_6_−1,4−(Me_2_SiCH_2_PPh_2_)_2_}] (M = Na (**2**), K (**3**), Rb (**4**), Cs (**5**)). The reaction was performed *via* deprotonation of **1** using either elemental Na or the Lochmann‐Schlosser bases^[^ ^61^ ^]^ *n*−BuLi with either K−*tert*−butoxide, Rb‐*tert*‐butoxide, or Cs−*tert*−butoxide (Scheme 2). The protons at the 1,4‐position of the COT, which are in *α*−position to silicon(IV), exhibit enhanced acidity relative to other COT protons, facilitating selective deprotonation with alkali metals.

While the sodium complex **2** could be obtained directly from elemental sodium, attempts to synthesize complexes **3**, **4**, and **5** using elemental potassium, rubidium, and cesium, respectively, resulted in ligand degradation due to the high reactivity of these metals down the group. To address this, an equimolar ratio (2:2) of M−*tert*−butoxide (M = K for **3**, M = Rb for **4**, and M = Cs for **5**) and *n*−BuLi were treated with one equivalent of the proligand **1** in *n*‐heptane to obtain [K_2_{C_8_H_6_−1,4−(Me_2_SiCH_2_PPh_2_)_2_}] (**3** reddish powder), [Rb_2_{C_8_H_6_−1,4−(Me_2_SiCH_2_PPh_2_)_2_}] (**4** orange powder) and [Cs_2_{C_8_H_6_−1,4−(Me_2_SiCH_2_PPh_2_)_2_}] (**5** reddish brown powder), respectively.^[^ ^24^, ^43^ ^]^ Complexes **3−5** exhibited good solubility in benzene, toluene, and polar solvents (THF and DME), in contrast to sodium complex **2**, which has poor solubility in these solvents. Complexes **2−5** demonstrated high sensitivity to air and moisture but remained stable at room temperature for several months under strictly anhydrous conditions. Surprisingly, attempts to synthesize the corresponding Li salt were unsuccessful. Elemental lithium exhibited no reactivity under the applied conditions, and the use of *n*−BuLi resulted in the formation of an oily, unidentifiable mixture, and isolation of a discrete product was not possible.

Compounds **2−5** were thoroughly characterized by single−crystal X−ray diffraction (SC−XRD), NMR spectroscopy, IR spectroscopy, and elemental analysis. Coordination of the phosphine groups to the alkali metal centers was confirmed by ^31^P{^1^H} NMR spectroscopy, which showed singlets at *δ* = −17.6 (**2**), −19.2 (**3**), −18.5 (**4**), and −17.5 ppm (**5**) (Figures S6, S10, S14, S18), representing consistent downfield shifts relative to the free ligand **1** (*δ* = −22.8 ppm, Figure S3). In contrast to the free ligand (*δ* = 5.2 ppm), the ^29^Si{^1^H} NMR spectra of complexes **2–5** revealed significant upfield shifts (*δ* = −0.5 ppm (**2**) to *δ* = −1.4 ppm (**5**)) (Figures S7, S11, S15, and S19, respectively). The ^29^Si{^1^H} NMR signals are systematically shifted toward the upfield region with the increasing size of the alkali metal, supporting metal−ligand interaction.

**Figure 2:**
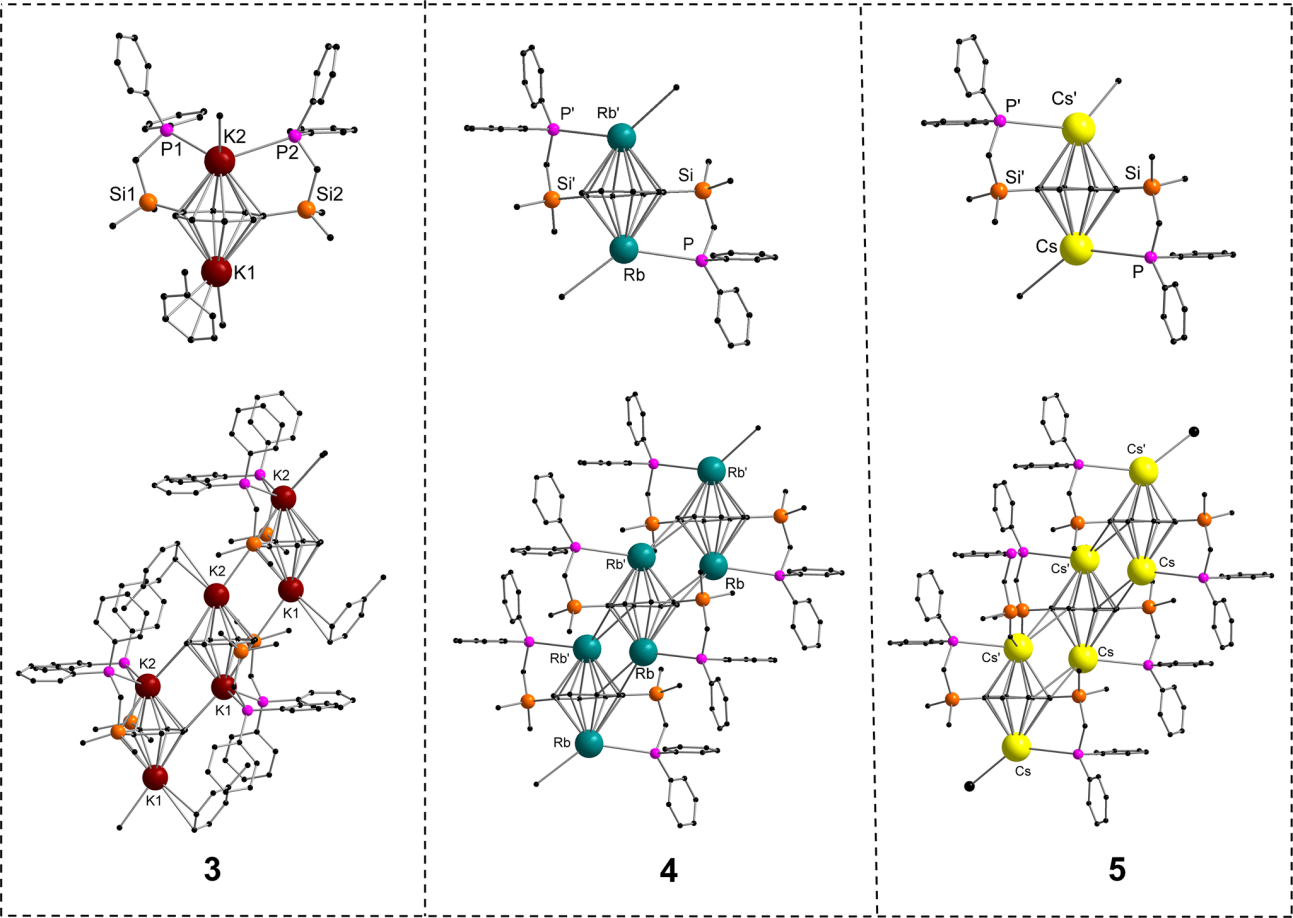
Molecular structures of [{K_2_{C_8_H_6_−1,4−(Me_2_SiCH_2_PPh_2_)_2_}(toluene)}_∞_] (**3**), [{Rb_2_{C_8_H_6_−1,4−(Me_2_SiCH_2_PPh_2_)_2_}_∞_] (**4**) and [{Cs_2_{C_8_H_6_−1,4−(Me_2_SiCH_2_PPh_2_)_2_}_∞_] (**5**) in the solid state. Hydrogen atoms and noncoordinating solvents are removed for clarity. Comprehensive data collection for these structures is provided in Supporting Information.

Compound **2** was crystallized by diffusion of *n*‐pentane into a THF solution, which yielded a solvent‐free inverse sandwich 1D polymeric structure (Figure S24). Due to the poor‐quality crystal data, we refrain from discussing the bonding parameter of **2**. However, the structure of **2** was established unambiguously from the difference Fourier map. The potassium complex **3**, which was crystallized from layering toluene with *n*‐hexane and isolated as a 1D polymeric structure (Figure 2), in which the COT ligand adopts a highly symmetric *μ*−*η* ^8^:*η* ^8^−bridging coordination mode. As a result, an “inverse sandwich” structure, which can be likened to previously reported COT‐based alkali metal complexes, was formed.^[^ ^17^, ^38^, ^51^, ^62^ ^]^ Each K metal ion is coordinated to COT in an *η* ^8^−mode. Furthermore, K2 is bound to both phosphine groups, while the coordination sphere of K1 is saturated by an *η* ^2^−coordinated cooridinated toluene molecule. Each K metal ion exhibits intermolecular *η* ^2^−coordination to a neighboring COT unit that facilitates the formation of a ladder‐like 1D polymeric architecture. The K−(*η*−COT) coordination distances are 3.134(2) Å and 3.208(8) Å.

The K2−P1 bond length is 3.429(14) Å, marginally longer than the K2−P2 bond, which is 3.388(13) Å. Analysis of the K−C interactions within the K(*η* ^8^−COT) framework reveals slight variations between the two potassium centers: K2(*η* ^8^−COT) bond lengths span from 2.895(2) to 3.048(2) Å, whereas K1−(*η* ^8^−COT) distances range from 2.923(2) to 3.014(2) Å. These values show only minor deviations and are consistent with previously reported potassium COT complexes, such as [{K_2_(C_8_H_6_−1,3,6−(SiMe_3_)_3_)(DME)_2_}_∞_],^[^ ^63^ ^]^ [K_2_{C_8_H_6_−1,4−(Si*^t^* BuMe_2_)_2_}(DME)_2_],^[^ ^62^ ^]^ and [K_2_(C_8_H_6_−1,4−(SiPh_3_)_2_)(DME)_4_].^[^ ^38^ ^]^ Consequently, it can be inferred that substitution of the COT ligand with various silyl groups exerts minimal influence on the K−COT coordination distances. Additionally, the interaction between K1 and the coordinated toluene molecule is defined by K1−(*η* ^2^−toluene) bond lengths ranging from 2.292(3) to 3.509(3) Å.

Complexes **4** and **5**, which exhibit isostructural solid‐state architectures, were crystallized from toluene or from toluene layered with *n*‐pentane. Both compounds form 1D solvent‐free polymeric structures, in which the COT ring adopts a highly symmetric *μ−η* ^8^:*η* ^8^‐COT bridging coordination mode (Figure 2). As a result, an inverse sandwich arrangement is formed in which each metal atom is *η* ^8^‐coordinated by COT and one phosphine moiety. As observed for complex **3**, each metal center in complexes **4** and **5** engaged in an intermolecular *η* ^2^‐coordination with a neighboring COT unit, which facilitates the formation of a ladder−like 1D polymeric architecture. While Rb−*η^2^*‐COT distances increase to 3.451(5) Å and 3.599(5) Å, consistent with the larger ionic radius of Rb. For the Cs analogue, *η* ^2^‐interactions persist with COT units, with extended bond lengths of 3.567(2) Å and 3.639(2) Å. The progressive elongation of metal‐ligand distances across the series K < Rb < Cs aligns with the expected trend based on increasing ionic radii. The other variation in the bonding pattern of complexes **4** and **5** and complex **3** can be attributed to the solvent used for crystallization. In complex **3**, the ligand adopts a symmetric coordination mode where both phosphines coordinate to a single potassium center. In contrast, complexes **4** and **5** exhibit a twisted geometry where the two phosphines coordinate to two different rubidium and cesium centers, respectively. This indicates the degree of structural flexibility and adaptability of the ligand in response to the size and coordination preferences of the metal ions.

The Rb−P bond length in **4** is 3.638(14) Å, which is slightly longer compared to the K−P distances in compound **3** (Figure 2). Furthermore, the Rb−C distances for the Rb−(*η* ^8^−COT) coordination reveal bond distances ranging from 3.148(5) to 3.073(5) Å, which is slightly longer compared to **3** (Figure 2). In **4**, the solvent has a significant influence on the structural outcome. Crystallization from toluene leads to the formation of a solvent‐free polymeric structure **4**.

In the cesium complex **5**, the Cs−P bond distance (Cs−P: 3.742(7) Å is predictably longer than the Rb−P bond distance in **4**, consistent with the larger ionic radius of cesium (Figure 2). The Cs−C distances to the *η^8^*−COT ring vary between 3.169(5) Å and 3.296(3) Å, slightly larger than in the Rb analogue **4** but maintaining a symmetrical coordination environment.

**Scheme 3:**
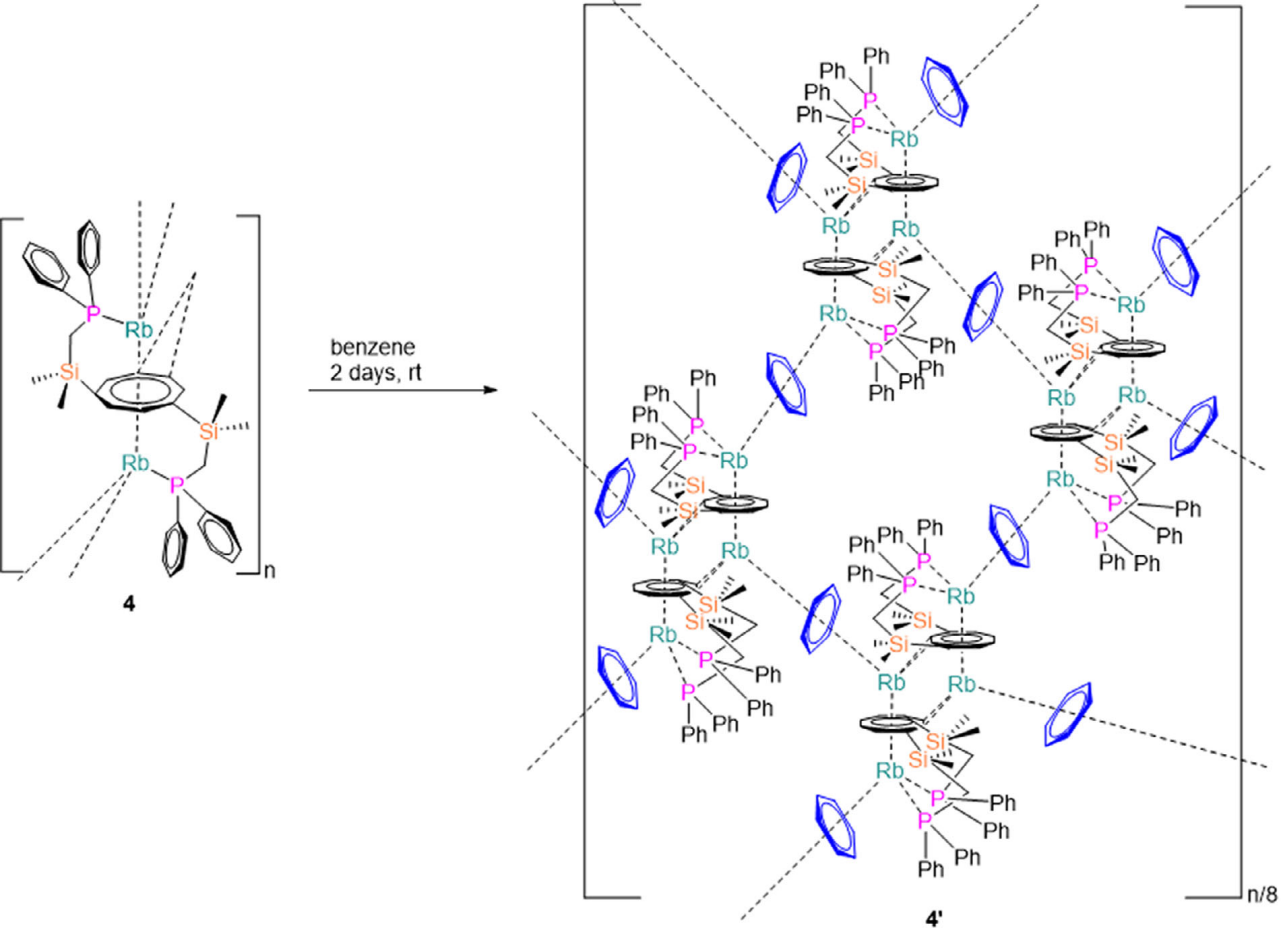
Synthesis of Rb complex [{Rb_2_(C_8_H_6_−1,4−(Me_2_SiCH_2_PPh_2_)_2_) (C_6_H_6_)_2_}_8_]_∞_ (**4′**).

**Figure 3:**
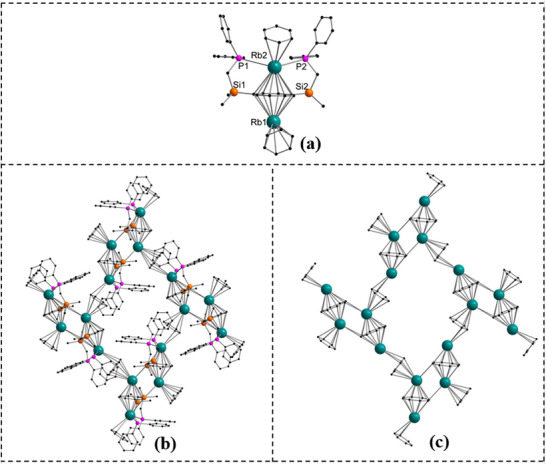
Molecular structure of **4′** in the solid state. a) Asymmetric unit, b) polymeric 8‐member ring, c) omitted substitution group of COT for clarity of the 8‐membered ring. Hydrogen atoms and noncoordinating solvents are removed for clarity. Comprehensive data collection for these structures is provided in Supporting Information.

When compound **4** was recrystallized from benzene (Scheme 3), it gave rise to a benzene‐bridged eight‐membered polymeric structure [{Rb_2_(C_8_H_6_−1,4−(Me_2_SiCH_2_PPh_2_)_2_) (C_6_H_6_)_2_}_8_]_∞_ (**4′**; Figure 3). In the molecular structure in the solid state, **4′** depicts two distinct coordination environments at the Rb ions. One Rb atom (Rb2) is coordinated to a planar *η* ^8^−COT moiety, two phosphine donors, and an *η* ^3^‐cooridnated benzene molecule. In contrast, the second Rb center (Rb1) features coordination to an *η* ^8^−COT moiety, an *η* ^6^‐cooridnated benzene molecule, and engages an intermolecular *η* ^2^‐cooridnation to a neighboring COT unit. Due to the intermolecular Rb1−COT *η* ^2^−interactions, the dimeric structure motifs of the {Rb_2_(C_8_H_6_−1,4−(Me_2_SiCH_2_PPh_2_)_2_)} units are formed. Four of these dimeric pairs are bridged by benzene molecules, forming the eight‐membered ring structure, but only eight out of the sixteen Rb forms are linked to form a ring (Figures 3b, c), while the other Rb ions are part of other adjacent rings, giving rise to the coordination polymer.

In **4′**, the Rb2−P1 and Rb2−P2 bond distances are 3.496(2) Å and 3.490(2) Å, respectively, slightly shorter than the corresponding bond lengths observed in the solvent‐free rubidium structure **4**. Analysis of the Rb−COT interactions within the *μ*−*η^8^*:*η^8^*−COT coordination sphere reveals that the Rb−C bond distances range from 3.050(8) Å to 3.174(8) Å, with negligible disparity between the two Rb centers, underscoring a highly symmetric bonding environment.

**Scheme 4:**
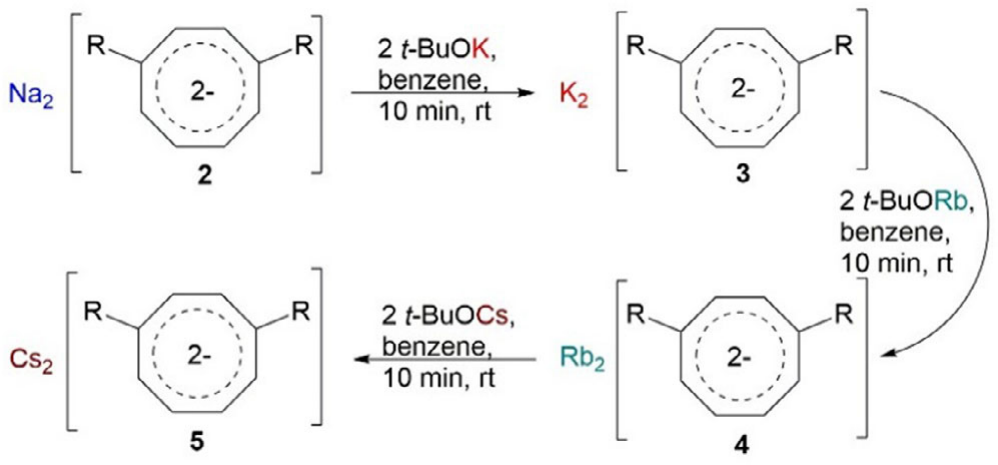
Synthesis of [M_2_{C_8_H_6_−1,4−(Me_2_SiCH_2_PPh_2_)_2_}] (M = K, Rb, Cs) *via* metal exchange reaction.

The reactivity of complexes **2**, **3**, and **4** with alkali metal *tert*‐butoxides was investigated on an NMR scale, providing an efficient alternative synthetic route to the alkali metal complexes **3**, **4**, and **5** (Scheme 4). These transformations were achieved under mild conditions with sonication at room temperature and reached completion within 10 minutes. ^31^P{^1^H} NMR spectroscopy showed conversion yields exceeding 99%. Obviously, the lighter alkali metal ion can be easily replaced by its heavier congener. These results represent an attractive alternative to traditional synthetic methods, which often require prolonged reaction times and more demanding reaction conditions.^[^ ^20^, ^24^, ^38^ ^]^

### 2.2 Photophysical Properties

The alkali metal complexes **2–5** were studied in terms of their photophysical properties in the solid state, both at 77 K and room temperature. The complexes exhibit almost no emission in solution, even at 77 K, likely because of solvent‐assisted nonradiative quenching. In the solid state, the onset of absorption in the PLE spectra at 460, 515, and 530 nm for complexes **2**, **4**, and **5** at room temperature correlates with their visual appearance and appears as yellow, orange, and red solids, respectively. On cooling the solid samples to 77 K, a blue shift of the onset of the absorption in the PLE spectra is observed for all the complexes.

**Figure 4:**
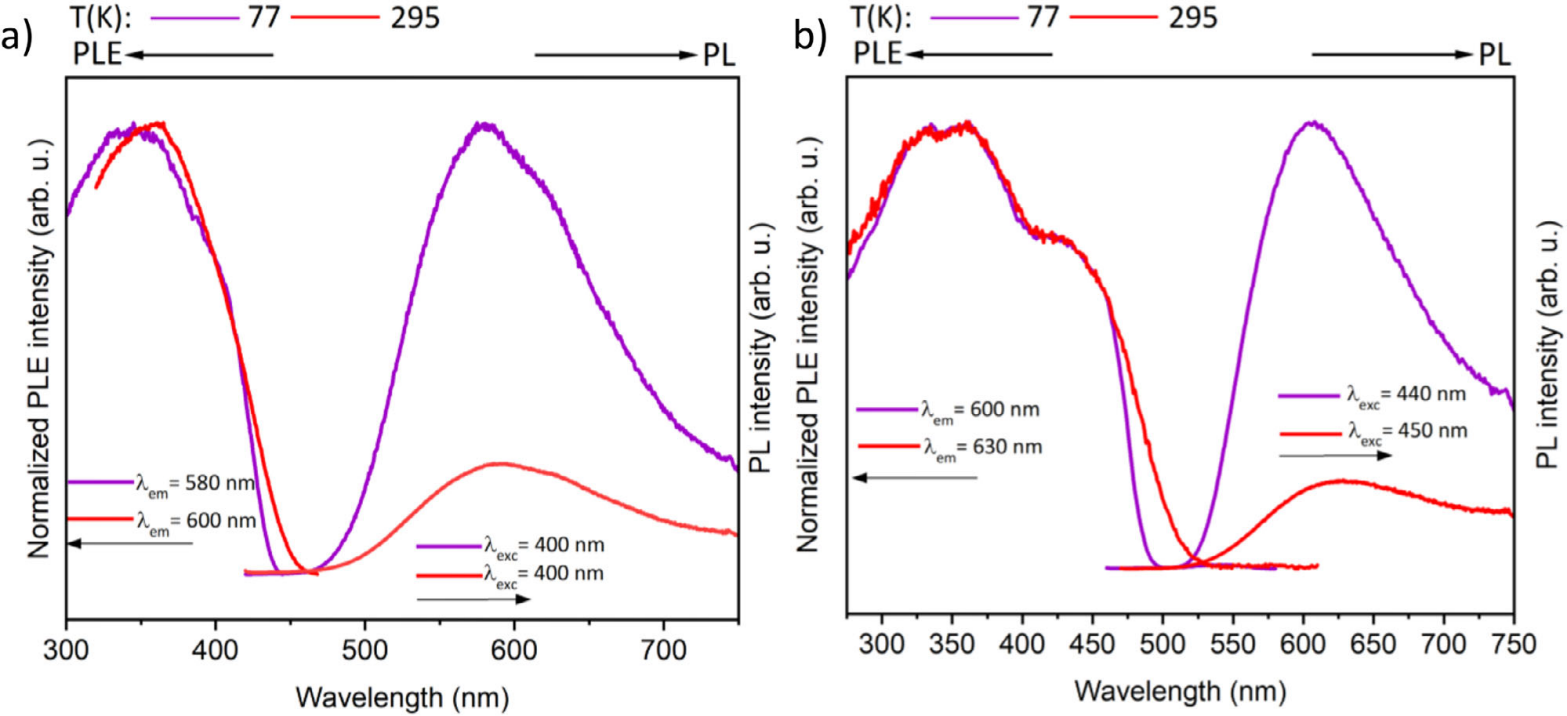
Photoluminescence excitation (PLE) and emission (PL) spectra of a) complex **2** and b) complex **5** in the solid state at 77 K and room temperature. PLE and PL spectra were recorded at the indicated emission and excitation wavelengths (*λ*_em_ and *λ*_exc_, respectively).

The sodium complex **2** shows a broad emission band centered at *λ*_max_ = 578 nm at 77 K (Figure 4a). The emission band is blue‐shifted to 580 nm on cooling the sample to 77 K. The excited states of the complex decay with a lifetime of 8.2 and 5.5 ns at 77 K and room temperature, respectively.

The potassium complex **3** practically shows no emission in the solid state, even on cooling down to 77 K. The nonemissive nature of the complex could be attributed to the coordination environment of the metal center (discussed below). The rubidium complex **4** shows a very weak emission band at 77 K centered at *c.a*. 613 nm. The excited state of the complex has a very short lifetime (<2 ns), which is below the detection limit of our detector.

Complex **5**, which has each metal center coordinated to a phosphine group, is again emissive both at 77 K and room temperature (Figure 4b). Complex **5** also has a broad emission like complex **2** with *λ*_max_ = 605 nm and 622 nm at 77 K and room temperature, respectively. On moving down the alkali metal group, a bathochromic shift of the emission band is observed. The lifetime of the excited state of complex **5** is determined to be 53.6 ns. Upon heating to room temperature, the lifetime is shortened to 3.7 ns due to enhanced nonradiative decay. A sixfold increase in the lifetime of the Cs complex is observed at 77 K when compared with the Na analogue. The lifetimes in the nanosecond region are indicative of the fluorescent nature of this series of complexes.

**Figure 5:**
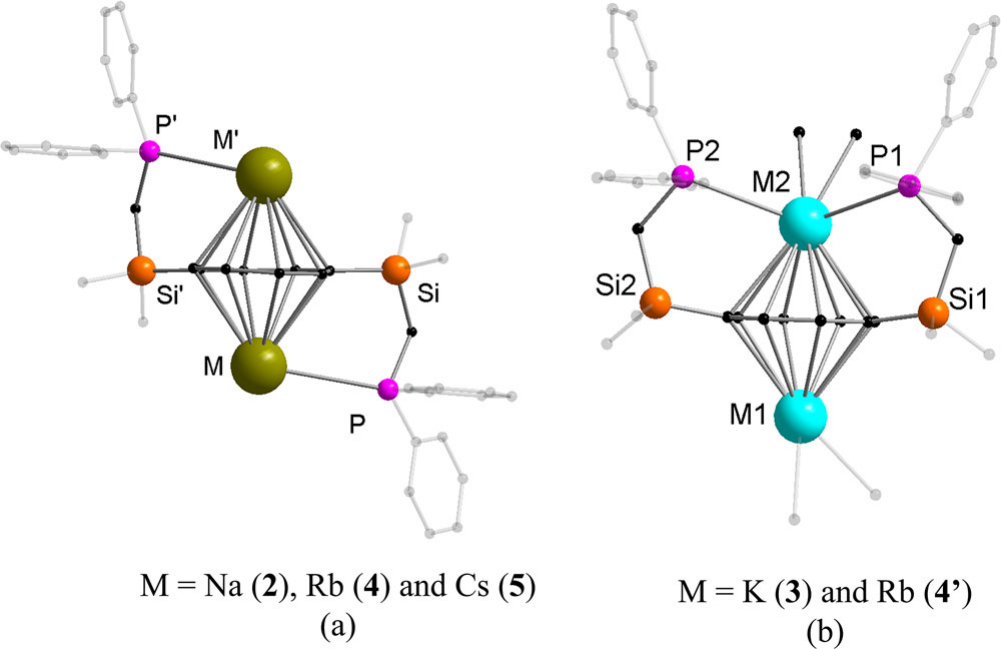
Coordination pattern of phosphine group and metal center: a) complexes **2**, **4**, and **5** and b) complexes **3** and **4′**. Hydrogen atoms and noncoordinating solvents are removed for clarity.

The different emissive behavior of the complexes can be attributed to the two different coordination modes observed among these series of compounds, which are illustrated in Figure 5. In complexes **2**, **4**, and **5** (Figures S24 and 5), the pendant phosphine groups of the ligand are coordinated to different metal centers. This results in a molecular structure where each metal center is bonded uniformly to a phosphine moiety (Figure 5b). Hence, the complexes are emissive. In contrast, both phosphine groups of the silyl‐substituted COT ring are coordinated to the same metal center in the asymmetric unit of the complexes **3** and **4′** (Figure 5b). This leads to the coordination of the second metal only to the COT ring and a solvent molecule. The lack of coordination by the phosphine moiety to one of the metals in the asymmetric unit leads to nonemissive behavior in complex **3**. The weak emissive nature of the Rb complex can also be tentatively attributed to the possibility of two different configurations in the solid state (Figures 2 and 5).

## 3 Conclusion

In summary, we have successfully synthesized and characterized a novel phosphine−functionalized COT ligand, {C_8_H_8_−1,4−(Me_2_SiCH_2_PPh_2_)_2_} as a precursor. The COT ligand unveiled its coordination behavior with alkali metals (Na, K, Rb, Cs), which resulted in the successful isolation and structural characterization of a complete series of metal complexes. Structural analyses reveal that the bonding mode is influenced by both the nature of the metal center and crystallization conditions. Notably, a unique eight‐membered Rb‐ring stabilized by *η^3^* and *η^6^* benzene interaction is reported, highlighting the potential of ligands to form intricate architectures. Beyond mere structural elucidation, incorporating pendant phosphine groups into the ligand framework is essential for geometric rigidity at the metal center and found to be a key factor in achieving luminescent behavior in these complexes. Additionally, metal exchange reactions with alkali metal *tert*‐butoxides established a rapid and efficient synthetic route for accessing these complexes. Therefore, we hope that our finding of phosphine‐functionalized COT ligands will provide structural diversity and open a new avenue for designing and utilizing phosphine‐functionalized polycyclic systems in coordination chemistry.

## Supporting Information

The authors have cited additional references within the Supporting Information.^[^ ^60^, ^64^, ^65^, ^66^, ^67^, ^68^, ^69^ ^]^ Data for this paper are available at radar4chem [https://radar.products.fiz‐karlsruhe.de/] at https://doi.org/10.22000/v6xz4wsadaj3tjk4.

## Conflict of Interest

The authors declare no conflict of interest.

## Supporting information



Supplementary Information

## Data Availability

The data that support the findings of this study are available in the supplementary material of this article. Data for this paper are available at radar4chem [https://radar.products.fiz−karlsruhe.de/] at https://doi.org/10.22000/10.22000/v6xz4wsadaj3tjk4.
