# Supplementary material for: Alkali Metal Complexes of a Phosphine‐Functionalized Cyclooctatetraene
Source: Chemistry. 2025 Aug 11;31(55):e02313. doi: 10.1002/chem.202502313 (PMC12498079; doi:10.1002/chem.202502313)
Supplement: Supplementary file 1 — Supplementary Information [file CHEM-31-e02313-s001.pdf]

Supporting Information

Alkali Metal Complexes of a Phosphine-Functionalized Cyclooctatetraene

Mohd Iqbal,[a] Vanitha R. Naina,[a] Xiaofei Sun,[a] Shubham[a] and Peter W. Roesky*[a,b]

a. Institute of Inorganic Chemistry,
Karlsruhe Institute of Technology (KIT),
Kaiserstr 12, 76131, Karlsruhe, Germany.
E-mail: roesky@kit.edu

b. Institute for Nanotechnology,
Karlsruhe Institute of Technology (KIT),
Kaiserstr 12, 76131, Karlsruhe, Germany.

TABLE OF CONTENTS

1. GENERAL PROCEDURES S2

2. SYNTHETIC PART S3

2.1. Ligand Synthesis [C_8H_8-1,4-($Me_2SiCH_2PPh_2$)] (1) S3

2.2. Metal complexes S4

2.2.1 Synthesis of sodium complex [Na_2{C_8H_6-1,4-($Me_2SiCH_2PPh_2$)}] (2) S4

2.2.2 Synthesis of potassium complex [K_2{C_8H_6-1,4-($Me_2SiCH_2PPh_2$)}] (3) S5

2.2.3 Synthesis of rubidium complex [Rb_2{C_8H_6-1,4-($Me_2SiCH_2PPh_2$)}] (4) S6

2.2.4 Synthesis of cesium complex [Cs_2{C_8H_6-1,4-($Me_2SiCH_2PPh_2$)}] (5) S7

2.2.5 Alternate route for the synthesis of complexes 3, 4 and 5. ..... S7

3. NMR SPECTRA S9

4. IR SPECTRA S19

5. X-RAY CRYSTALLOGRAPHY STUDIES S21

6. PHOTOLUMINESCENCE MEASUREMENTS S28

7. REFERENCES S30

1. General procedures

All reactions were carried out with rigorous exclusion of air and water in oven-dried glassware under an inert atmosphere of nitrogen and argon, employing standard Schlenk, high-vacuum, and glovebox techniques. All Solvents were dried using an *MBraun* solvent purification system (*SPS-800*), except THF and hexanes underwent purification by drying over Na/benzophenone followed by distillation prior to use. The deuterated solvent C_6D_6 was dried over NaK alloy, vacuum-transferred and degassed before use. Cyclooctatetraene was distilled and degassed before used, other chemicals, methyl diphenyl phosphine, *n*-BuLi (1.6 M/2.5 M), *tert*-butanol, sodium, potassium, potassium *tert*-butoxide, TMEDA, cesium and rubidium were obtained from commercial sources and used without further purification.

The precursors, ((chlorodimethylsilyl)methyl)diphenylphosphane, cesium *tert*-butoxide and rubidium *tert*-butoxide were synthesized following previously reported methods[1, 2] and stored in glovebox.

NMR spectra were recorded on Bruker spectrometers (Avance Neo 300 MHz, Avance Neo 400 MHz or Avance III 400 MHz). Chemical shifts are referenced internally using signals of the residual protio solvent (^{1}H) or the solvent ($^{13}C\{^{1}H\}$) and are reported relative to tetramethylsilane (^{1}H, $^{13}C\{^{1}H\}$), H_3PO_4 ($^{31}P\{^{1}H\}$), or or externally relative to tetramethylsilane (^{29}Si). All NMR spectra were measured at 298 K, unless otherwise specified. The multiplicity of the signals is indicated as s = singlet, d = doublet, dd = doublet of doublets, t = triplet, q = quartet, m = multiplet and br = broad. Assignments were determined based on unambiguous chemical shifts, coupling patterns and ^{13}C-DEPT experiments.

Elemental analysis were carried out with an Elementar Unicube. The compounds were prepared using tin boats in argon-filled glove boxes.

Infrared (IR) spectra were recorded in the region 4000–400 cm^{-1} on a Bruker Tensor *37* FTIR spectrometer equipped with a room temperature DLaTGS detector, a diamond attenuated total reflection (ATR) unit and a nitrogen-flushed chamber. In terms of their intensity, the signals were classified into different categories (vs = very strong, s = strong, m = medium, w = weak, and sh = shoulder). UV-Vis spectra were recorded on an *USB4000* spectrometer together with a *USB-ISS-UV-Vis* device from Ocean Optics. The samples were dissolved in THF and measured using a 1 cm path quartz cell sealed with a young valve. Measurements were conducted in single strobe mode, the holdoff time and pulse width were both set to 25 µs, the integration time was between 30 and 35 µs, and electric dark correction was turned on. At least 20 scans were averaged and smoothed employing a boxcar smoothing value between two and six. Both light sources were turned on with the UV lamp adjusted to 100 % and the visible lamp to 72 %, respectively. Spectra were collected from 210–800 nm and baseline corrected with respect to the pure solvent. The observed signals are classified into different categories (max = most intense signal, sh = shoulder, br = broad signal).

PL measurements were carried out on a PTI QuantaMasterTM 8075-22 fluorometer with double excitation and emission monochromators (HORIBA Jobin Yvon GmbH). The samples (polycrystalline solids) were each sealed under an inert atmosphere in NMR tubes with a J. Young valve (material

Suprasil® quartz glass). The tube was placed in a glass dewar vessel (equipped with a suprasil finger on the bottom where spectroscopy takes place) which was filled with liquid nitrogen for measurements at 77 K. For emission detection, a R928 photomultiplier (250–800 nm) (HORIBA Jobin Yvon GmbH) was used. All spectra were corrected for the wavelength-dependent response of the detector (in relative photon flux units) and the spectrometer. For detection of the emission decay traces, the sample was excited with either a Delta DiodeTM (HORIBA Jobin Yvon GmbH, Model DD-370, λ_{exc} = 371 nm, pulse <2 ns, 2 μW) for fluorescence lifetimes The signal was recorded until a satisfying signal-to-noise ratio was obtained.

2. Synthetic Part

2.1. Ligand Synthesis [C_8H_8-1,4-($Me_2SiCH_2PPh_2$)] (1)

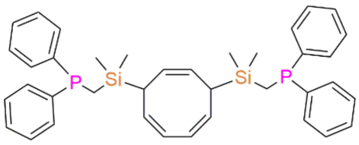

A piece of potassium (2 eq., 2 g, 51.15 mmol) was added in a round-bottom flask charged with THF (150 ml). The mixture was stirred and cyclooctatetraene (COT) (1 equiv. 2.66 g, 25.58 mmol) was added dropwise to the stirred solution at –78 °C. The reaction mixture was gradually warmed to room temperature, and stirred until the complete consumption of potassium, the mixture was filtered to remove any unreacted metal. The resulting filtrate was cooled again to –78 °C and ((chlorodimethylsilyl)methyl)diphenylphosphine (2 equiv. 14.98 g, 51.15 mmol) was introduced dropwise. The temperature was then allowed to return to ambient conditions, and the reaction mixture was stirred for an additional 16 hours. Subsequent removal of the solvent under reduced pressure afforded an oily residue. This residue was extracted with pentane, and the solvent was evaporated to yield the target compound, 1,4-bis(dimethylsilylmethyl)diphenylphosphinecycloocta-2,5,7-triene {C_8H_8-1,4-($Me_2SiCHPPh_2$) **1** was obtained as a brown oil.

Yield: 82% (13 g, 21.01 mmol)

^{1}H NMR (400 MHz, C_6D_6): δ [ppm] = 7.58-7.55 (m, 10H, Ph-*H*) 7.14-7.09 (m, 10H Ph-*H*), 5.98-5.95 (m, 2H, C*H*-COT), 5.60-5.56 (m, 4H, C*H*-COT), 2.98-2.94 (m, 2H, C*H*-COT), 1.49 (s, 4H, -CH_2-), 0.07 (s, 12 H, Si(CH_3)).

^{13}C{^{1}H} NMR (101 MHz, C_6D_6): δ [ppm] = 141.9 (d, J_{CP} = 5.5 Hz, Ph), 133.01 (d, J_{CP} = 19.8 Hz, Ph), 128.5 (d, J_{CP} = 4.0 Hz Ph), 128.4 (COT), 126.5 (COT), 123.9 (COT), 35.27 (d, J_{CP} = 4.4 Hz, COT), 13.31 (d, J_{CP} = 31.2 Hz) (-CH_2-), -1.86 (d, J_{CP} = 4.8 Hz, Si(CH_3)).

^{29}Si{^{1}H} NMR (80 MHz, C_6D_6): δ [ppm] = 5.2 (d, J_{SiP} = 15.2 Hz).

^{31}P{^{1}H} NMR (162 MHz, C_6D_6): δ [ppm] = -22.1 (s)

2.2. Metal complexes

2.2.1 Synthesis of sodium complex [$Na_2\{C_8H_6$-1,4-($Me_2SiCH_2PPh_2$)}] (2)

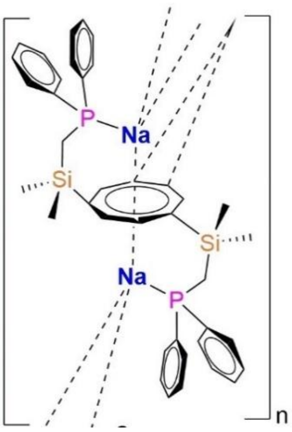

A mixture of compound **1** (1 equiv., 1.00 g, 1.62 mmol) and sodium metal (4 equiv., 148 mg, 6.46 mmol) was placed in a 100 mL Young flask. To this, *n*-heptane (50 mL) and THF (10 mL) were added at room temperature under stirring. After 1 hour, the reaction mixture gradually turned brown, and the formation of a precipitate was observed. The resulting suspension was stirred continuously at room temperature for an additional 12 hours. The precipitate was then collected by filtration and washed thoroughly with *n*-heptane until the washings became colorless. After drying under reduced pressure, the product **2** [Na_2COT-($Me_2SiCHPPh_2$)] was isolated as a yellow powder.

Single crystals suitable for X-ray were obtained by THF and pentane diffusion, and small needle like crystals formed after 5 days at ambient temperature.

Note: No coordinated solvent was detected in NMR spectra and elemental analysis.

Yield: 60%. (598 mg, 969.4 mmol)

Elemental analysis [%] calculated for [$C_{38}H_{42}Na_2Si_2P_2$]: C 68.86, H 6.39; found: C 68.66, H 6.27.

^{1}H-NMR (400. MHz, C_6D_6): δ [ppm] = 7.30-7.27 (m, 10H, Ph-*H*), 7.09-7.02 (m, 10H, Ph-*H*, 6.71-6.67 (m, 4H, C*H*-COT), 6.51-6.48 (m, 2H, C*H*-COT), 1.58 (d, $^2J_{HP}$ = 5.1 Hz, 4H, -CH_2-), 0.58 (s, 12H, Si(CH_3)).

^{13}C{^{1}H} NMR (No NMR recorded because of bad solubility)

^{29}Si{^{1}H} NMR (80 MHz, C_6D_6): δ [ppm] = -0.59 (d, J_{SiP} = 15.1 Hz).

^{31}P{^{1}H} NMR (162 MHz, C_6D_6): δ [ppm] = -17.6 (s).

IR (ATR): $\tilde{\nu}$ (cm^{-1}) = 3070 (w), 3051 (w), 2998 (w), 2901 (w), 2169 (w), 2118 (w), 2081 (w), 2004 (w), 1974 (w), 1479 (m), 1432 (s), 1245 (w), 1101 (w), 1076 (m), 1060 (m), 1026 (m), 987 (s), 948 (s), 927 (w), 833 (w), 863 (m), 825 (w), 815 (vs), 779 (s), 758 (s), 735 (vs), 719 (s), 692 (vs), 670 (s), 621 (m), 577 (m), 507 (s), 470 (m), 425 (w).

Due to the limited solubility of complex **2**, various crystallization techniques were explored. However, successful crystallization was achieved only through slow diffusion of *n*-pentane into THF solution. Despite obtaining single crystals suitable for X-ray diffraction, the crystal data quality was suboptimal, giving only a basic molecular structure (Figure S24) without sufficient crystallographic data to support a detailed discussion. Some specific details are given below.

Crystal system	Monoclinic
**Space group**	*C2/c*
***a*/Å**	19.114(7)
***b*/Å**	25.235(13)
***c*/Å**	8.973(4)
***β*/°**	112.96(3)°
***V*/Å^3**	3986(3)

2.2.2 Synthesis of potassium complex [$K_2\{C_8H_6$-1,4-($Me_2SiCH_2PPh_2$)}] (3)

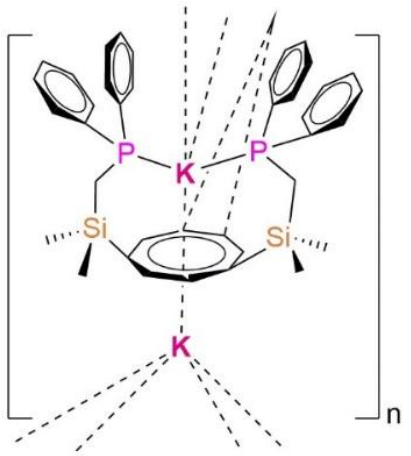

n-BuLi (1.6 M in hexanes, 2 equiv., 2.00 mL, 3.23 mmol) was added dropwise to a stirred suspension of compound **1** (1 equiv., 1.00 g, 1.62 mmol) and potassium *tert*-butoxide (2 equiv., 362 mg, 3.23 mmol) in *n*-heptane (150 mL), contained in a 250 mL young flask at room temperature. Upon completion of the addition, the reaction mixture turned deep red, and the formation of deep red precipitates was observed. The resulting suspension was stirred for an additional 12 hours at ambient temperature. The precipitates were subsequently collected by filtration and washed thoroughly with *n*-heptane until the washings became colorless. After drying under reduced pressure **3** [K_2COT-($Me_2SiMePPh_2$)] was obtained as a reddish powder. Upon drying, the coordinated solvent molecules were easily removed.

Single crystals suitable for X-ray crystallographic analysis were obtained by layering a toluene solution of the product with *n*-pentane. After standing undisturbed for five days at room temperature, block-shaped crystals were formed.

Note: No coordinated solvent was detected in NMR spectra and elemental analysis.

Yield: 86% (970 mg, 1.40 mmol).

Elemental analysis [%] calculated for [$C_{38}H_{42}K_2Si_2P_2$]: C 65.67, H 6.09; found: C 65.09, H 6.01.

^{1}H NMR (400. MHz, C_6D_6): δ [ppm] = 7.53-7.50 (m, 10H, Ph-*H*), 7.18-7.06 (m,10 H, Ph-*H*), 6.72-6.56 (m, 6H, C*H*-COT), 1.78 (d, $^2J_{HP}$ = 4 Hz, 4H, -CH_2-), 0.67 (s 12H, Si(CH_3)$_2$),

^{13}C{^{1}H} NMR (101 MHz, C_6D_6): δ [ppm] = 142.5 (d, J_{CP} = 11.0 Hz), 133.2 (d, J_{CP} = 18.7 Hz, Ph), 128.4 (d, J_{CP} = 6.6 Hz, Ph), 128.3 (COT), 97.7 (d, J_{CP} = 11.7 Hz), 94.2 (COT), 88.4 (COT), 19.4 (d, J_{CP} = 22.7 Hz, -*C*H_2-), 1.7 (d, J_{CP} = 3.7 Hz, Si(*C*H_3)$_2$).

^{29}Si{^{1}H} NMR (80 MHz, C_6D_6): δ [ppm] = -0.61 (d, $^2J_{SiP}$ = 15.2 Hz).

^{31}P{^{1}H} NMR (162 MHz, C_6D_6): δ [ppm] = -19.2 (s).

IR (ATR): $\tilde{\nu}$ (cm^{-1}) = 3063 (w), 3052(w), 3025 (w), 3008 (m), 2998 (m), 2944 (w), 2909 (w), 2897 (w), 2862 (w), 2216 (w), 2177 (w), 2150 (w), 2127 (w), 2101 (w), 2062 (w), 2050 (w), 2039 (w), 2012 (w), 1992 (w), 1972 (w), 1943 (w), 1579 (w), 1544 (w), 1524 (w), 1491 (w), 1479 (m), 1431 (m), 1364 (w), 1327 (w), 1301 (w), 1243 (m), 1207 (w), 1155 (w), 1099 (m), 1077 (m) 1061 (w), 1025 (m), 997 (w), 975 (m), 924 (m), 906 (w), 864 (w), 816 (s), 775 (m), 739 (s), 695 (vs), 674 (w), 619 (w), 569 (w), 509 (m), 472 (w), 423 (w),

2.2.3 Synthesis of rubidium complex [$Rb_2\{C_8H_6$-1,4-($Me_2SiCH_2PPh_2$)\}] (4)

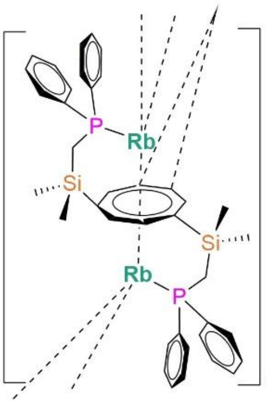

n-BuLi (2.5 M in hexanes, 2 equiv., 1.29 mL, 3.23 mmol) was added dropwise to a stirred suspension of **1** (1 equiv., 1.00 g, 1.62 mmol) and rubidium *tert*-butoxide (2 equiv., 512 mg, 3.23 mmol) in *n*-heptane (150 mL) contained in a 250 mL Young flask at room temperature. Upon complete addition, the reaction mixture developed a deep red coloration, and the formation of yellow precipitates was observed. The suspension was stirred for an additional 12 hours at ambient temperature. The resulting precipitates were collected by filtration and thoroughly washed with *n*-heptane until the washings were colorless. After drying under reduced pressure **4** [Rb_2COT-($Me_2SiCHPPh_2$)] was obtained as an orange powder.

Single crystals suitable for X-ray diffraction were obtained via slow concentration of a toluene solution or toluene layered with *n*-pentane or obtained *via* slow concentration of a toluene solution in this procedure, the solution was gently heated and then allowed to stand at room temperature, leading to the formation of bright yellow crystals of the solvent-free structure **4** within 2 hours. Using benzene as the solvent and applying the same crystallization procedure, crystal growth required three to four days. In this case, an eight-membered cyclocene-like structure with bridging benzene molecules **4'** was formed. Upon drying, the coordinated solvent molecules were easily removed.

Note: No coordinated solvent was detected in NMR spectra for complex **4'** and elemental analysis.

Yield: 75% (955 mg, 121 mmol).

Elemental analysis [%] calculated for [$C_{38}H_{42}Rb_2Si_2P_2$]: C 57.94, H 5.37, found: C 57.37, H 5.18.

^{1}H NMR (400. MHz, C_6D_6): δ [ppm] = 7.30-7.27 (m, 10H, Ph-*H*$_2$), 7.09-7.02 (m,10H, Ph-*H*), 6.71-6.67 (m, 4H, C*H*-COT), 6.51-6.48 (m, 2H, C*H*-COT), 1.85 (d, $^2J_{HP}$ = 3.7 Hz, 4H, -C*H*$_2$-).0.58 (s 12H, Si (C*H*$_3$)$_2$).

^{13}C{^{1}H} NMR (101 MHz, C_6D_6): δ [ppm] = 143.44 (d, J_{CP} = 14.3 Hz), 133.2 (d, J_{CP} = 18.3 Hz, Ph), 128.41 (d, J_{CP} = 6.6 Hz, (Ph), 128.3 (COT), 98.9 (d, J_{CP} = 18.7 Hz), 95.3 (COT), 89.9 (COT), 19.4 (d, J_{CP} = 24.6 Hz, -*C*H$_2$-), 2.1 (d, J_{CP} = 4.0 Hz, Si(*C*H$_3$)$_2$).

^{29}Si{^{1}H} NMR (80 MHz, C_6D_6): δ [ppm] -1.18 (d, J_{SiP} = 15.7 Hz).

^{31}P{^{1}H} NMR (162 MHz, C_6D_6): δ [ppm] = δ -18.5 (s).

IR (ATR): $\tilde{\nu}$ (cm^{-1}) = 3064 (w), 304 7(w), 2998 (m), 2965 (m), 2941 (m), 2899 (m), 2856 (m), 2742 (w), 2159 (w), 2136 (w), 2092 (w), 2073 (w), 2042 (w), 1976 (w), 1886 (w), 1709 (w), 1690 (w), 1582 w), 1569 w), 1543 (w), 1478 (w), 1449 (m), 1430 (s), 1360 (m), 1305 (m), 1272 (w), 1238 (s), 1203 (w), 1181 (m), 1155 (w), 1098 (s), 1059 (w), 1023 (w), 977 (s), 922 (m), 863 (vs), 771 (vs), 740 (vs), 693 (vs), 671 (s), 614 (m), 563 (w), 508 m), 472 (w), 423 (w)

2.2.4 Synthesis of cesium complex [$Cs_2\{C_8H_6$-1,4-($Me_2SiCH_2PPh_2$)}] (5)

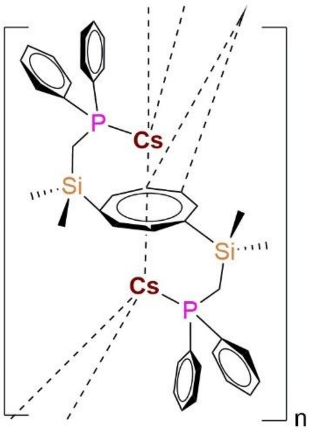

n-BuLi (2.5 M in hexanes, 2 equiv., 1.29 mL, 3.23 mmol) was added dropwise to a stirred suspension of **1** (1 equiv., 1.00 g, 1.62 mmol) and cesium *tert*-butoxide (2 equiv., 665 mg, 3.23 mmol) in n-heptane (150 mL), contained in a 250 mL Young flask at room temperature. Upon complete addition, the reaction mixture turned deep red, accompanied by the formation of yellow precipitates. The suspension was stirred for an additional 12 hours at ambient temperature. The resulting precipitate was collected by filtration and washed thoroughly with *n*-heptane until the washings became colorless. After drying under reduced pressure **5** [Cs_2COT-($Me_2SiCHPPh_2$)] was isolated as a reddish-brown powder.

Single crystals suitable for X-ray diffraction were obtained by freezing a toluene solution of the compound layering it with *n*-pentane and allowing it to stand at room temperature. After two days, large black-red crystals were formed. The supernatant was decanted and the crystals were washed with pentane.

Note: No coordinated solvent was detected in NMR spectra and elemental analysis.

Yield: 84%, (1.2 g, 136 mmol)

Elemental analysis [%] calculated for [$C_{38}H_{42}Cs_2Si_2P_2$]: C 51.71, H 4.80; found: C 52.15, H 4.96.

^{1}H NMR (400 MHz, C_6D_6): δ [ppm] 7.62-7.58 (m, 10H, Ph-*H*) 7.18-7.06 (m, 10H Ph-*H*) = 6.67-6.63 (m, 2H, C*H*-COT), 6.60-6.56 (m, 4H, C*H*-COT), 1.93 (d, J_{HP} = 3.3 Hz, 4H, -CH_2-), 0.63 (s 12H, Si (CH_3));

^{13}C{^{1}H} NMR (101 MHz, C_6D_6): δ [ppm] = 143.44 (d, J_{CP} = 14.3 Hz), 133.2 (d, J_{CP} = 18.3 Hz, Ph), 128.41 (d, J_{CP} = 6.6 Hz, Ph), 128.3 (COT) 100.72 (d, J = 7.0 Hz, (COT). 95.3 (COT), 89.9 (COT), 19.2 (d, J_{CP} = 24.6 Hz, -CH_2-), 2.4 (d,J_{CP} = 4.0 Hz, Si(CH_3)$_2$).

^{29}Si{^{1}H} NMR (80 MHz, C_6D_6) δ [ppm] -1.44 (d, J_{SiP} = 16.1 Hz).

^{31}P{^{1}H} NMR (162 MHz, C_6D_6, 298 K): δ [ppm] = -17.5 (s)

IR (ATR): $\tilde{\nu}$ (cm-1) = 3095 (w), 3065 (w), 3046 (w), 2996 (w), 2959 (w), 2931 (m), 2900 s), 2880 m), 2855 (w), 2242 w), 2187 (w), 2136 (w), 2083 (w), 2066 (w), 1977 (w), 1584 (w), 1546 (w), 1479 (m), 1430 (s), 1363 (w), 1326 (w), 1237 (s), 1196 (w), 1153 (w), 1101 (w), 1075 (s), 1057 (s), 1026 (w), 1000 (w), 973 (w), 928 (s), 863 (m), 815 (vs), 771 (vs), 736 (vs), 690 (vs), 675 (s), 616 (m), 556 (m), 524 (m), 504 (s), 467 (m), 422 w),

2.2.5 Alternate route for the synthesis of complex 3, 4 and 5.

Mixture of complex **2** (1 eq. 10 mg, 15.9 mmol) and potassium *tert*-butoxide (2 eq. 3.39 mg, 0.030 mmol) in C_6D_6 was prepared in an NMR tube by condensing the solvent at -78 °C and the reaction mixture was sonicated at room temperature for 10 minutes. Monitoring the reaction progress by $^{31}P\{^1H\}$ NMR spectroscopy revealed a complete conversion of complex **2** to complex **3**. Subsequently, under an inert atmosphere in a glovebox, rubidium *tert*-butoxide (2 eq. 4.78 mg, 0.030 mmol) was added to the same NMR tube containing complex **3**. The mixture was sonicated for another 10 minutes, and $^{31}P\{^1H\}$ NMR analysis confirmed the full conversion of complex **3** to complex **4**. Following the same procedure, the reaction of complex **4** with cesium *tert*-butoxide (2 eq. 6.22 mg, 0.030 mmol) resulted in the complete conversion to complex **5**, as evidenced by $^{31}P\{^1H\}$ NMR spectroscopy.

3. NMR spectra

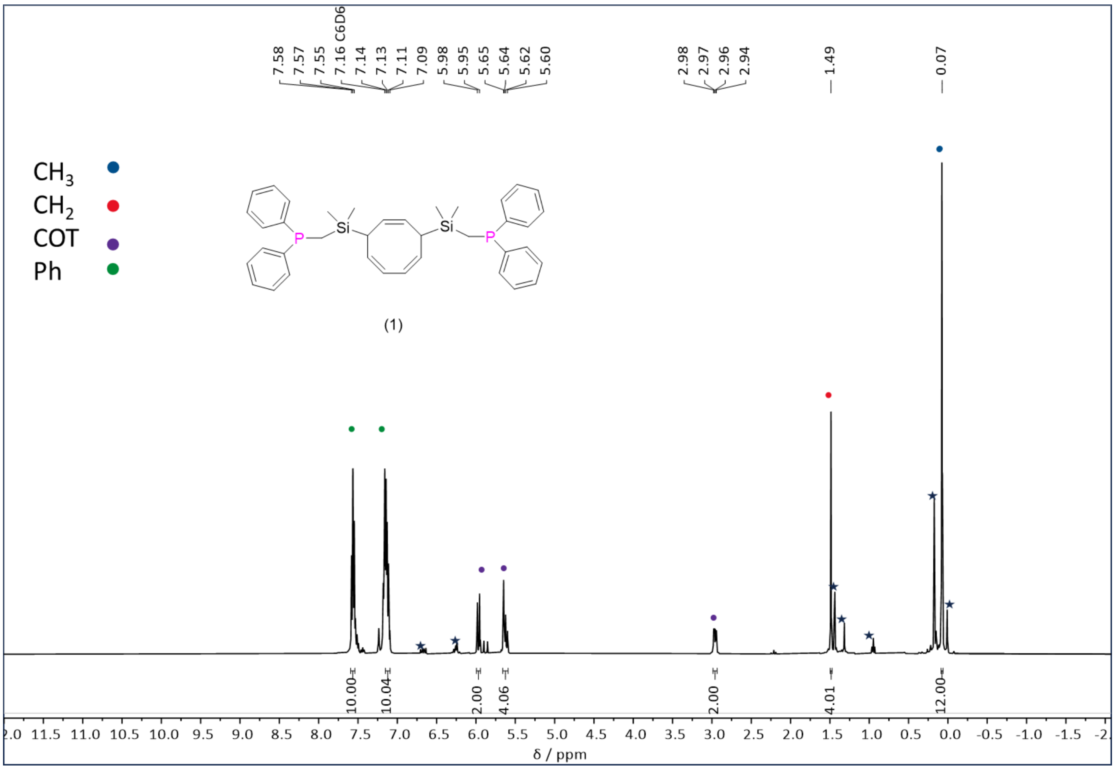

Figure S1: ^{1}H-NMR spectrum of **1** in C_6D_6 at room temperature. (★) denotes unidentified impurities.

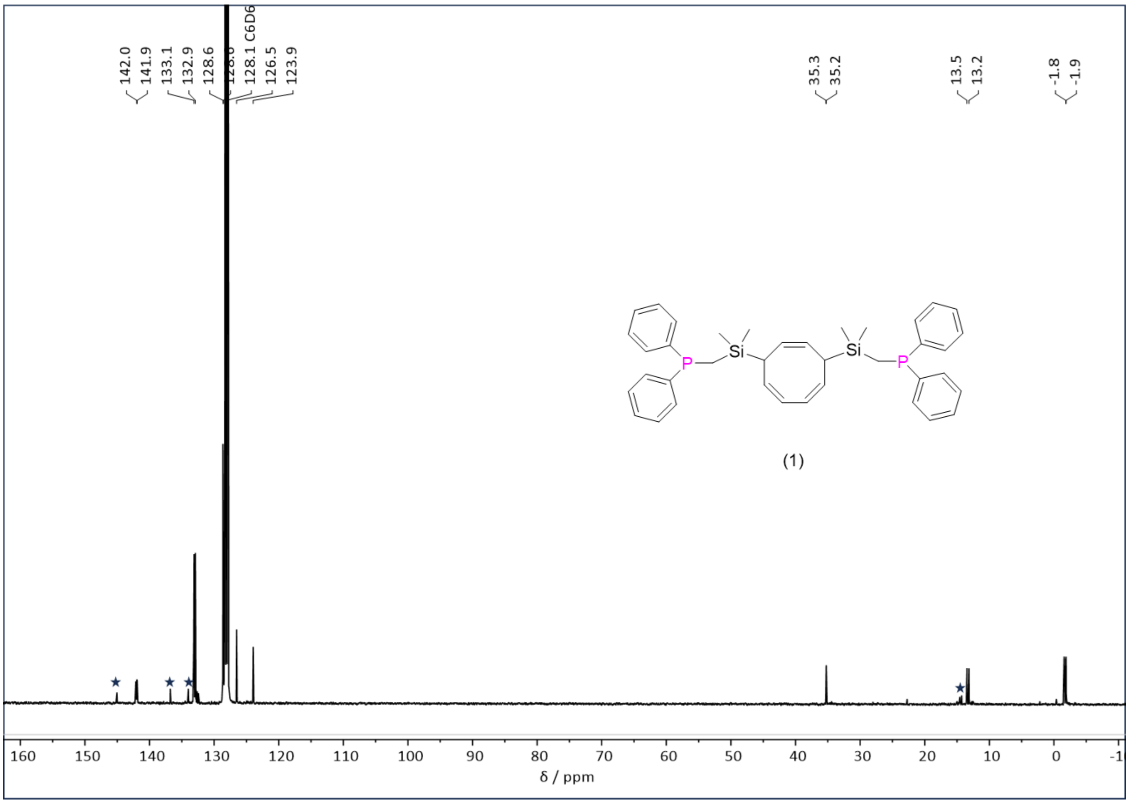

Figure S2: $^{13}C\{^{1}H\}$ NMR spectrum of **1** in C_6D_6 at room temperature. (★) denotes unidentified impurities.

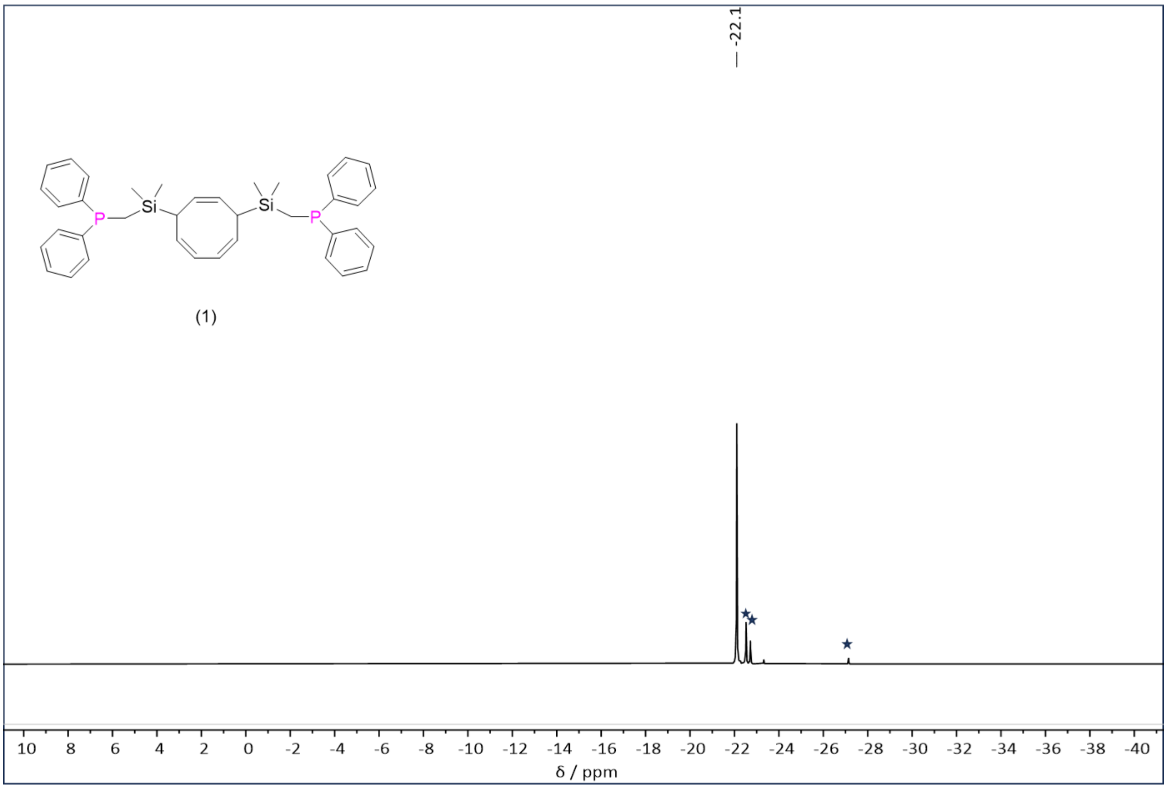

Figure S3: $^{31}P\{^{1}H\}$ NMR spectrum of **1** in C_6D_6 at room temperature. (★) denotes unidentified impurities.

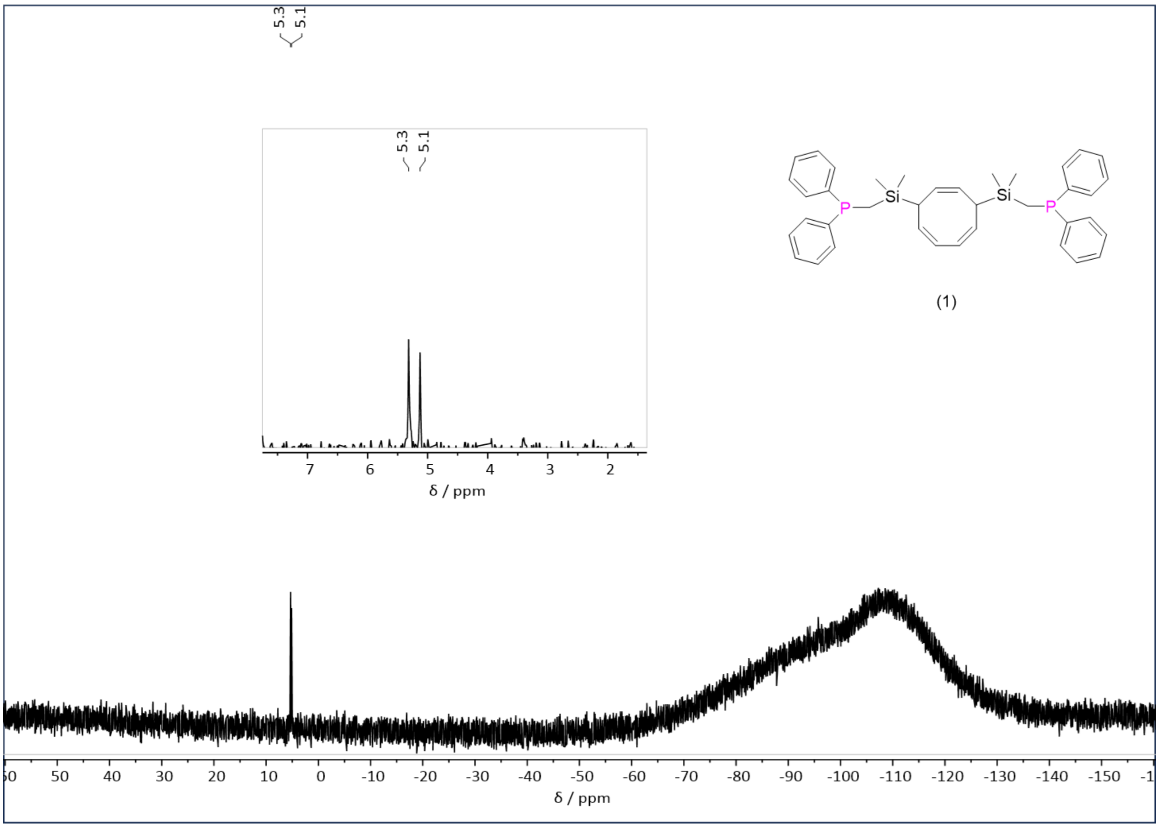

Figure S4: $^{29}Si\{^{1}H\}$ NMR spectrum of **1** in C_6D_6 at room temperature.

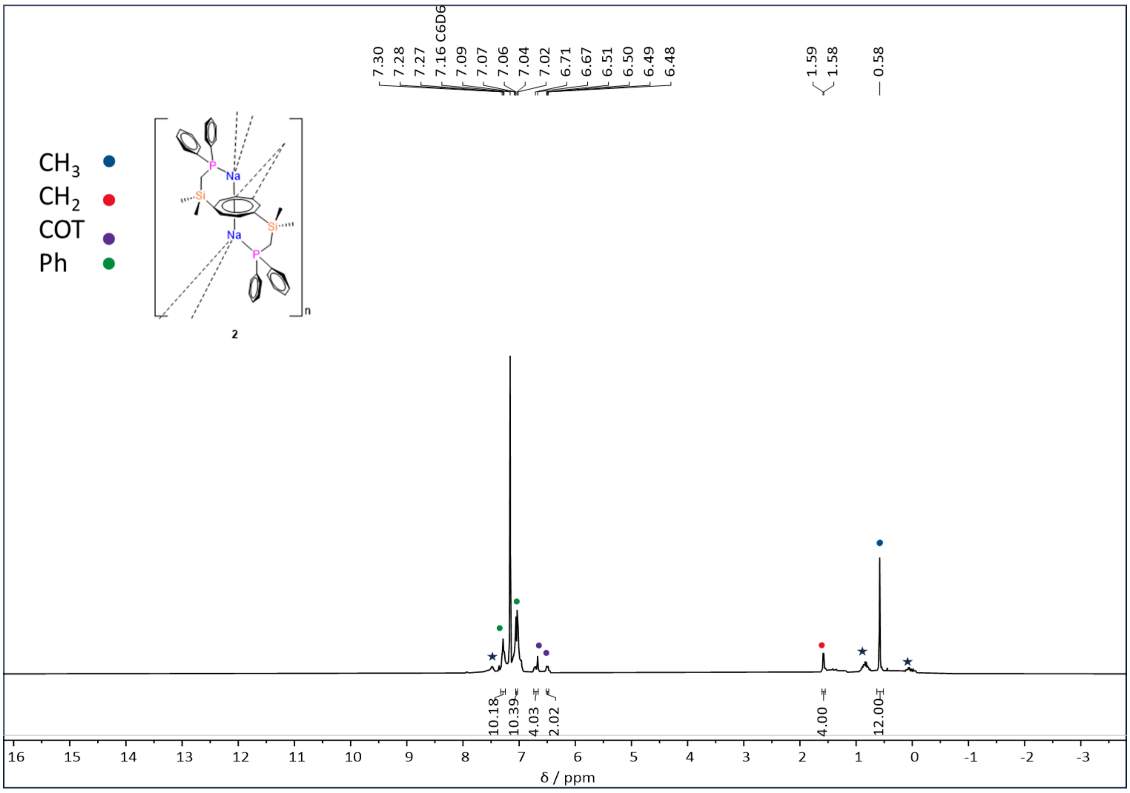

Figure S5: ^{1}H NMR spectrum of **2** in C_6D_6 at room temperature. (★) denotes unidentified impurities.

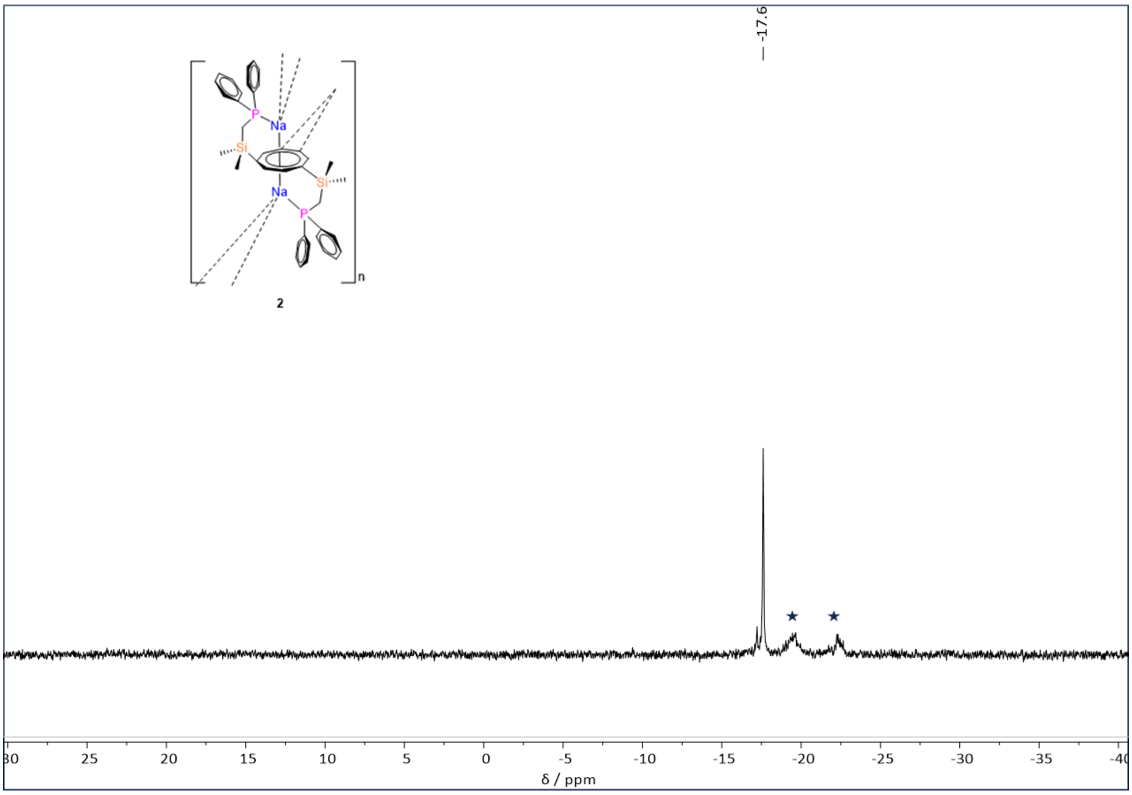

Figure S6: $^{31}P\{^1H\}$ NMR spectrum of **2** in C_6D_6 at room temperature. (★) unidentified impurities.

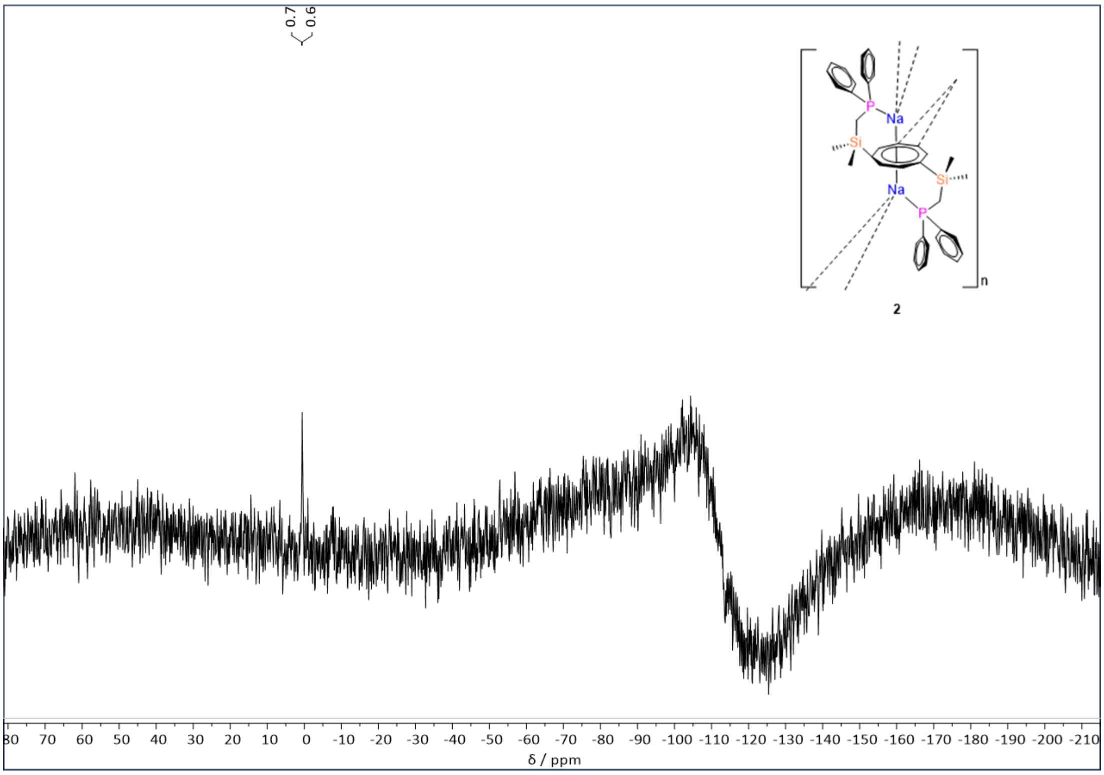

Figure S7: $^{29}Si\{^1H\}$ NMR spectrum of **2** in C_6D_6 at room temperature.

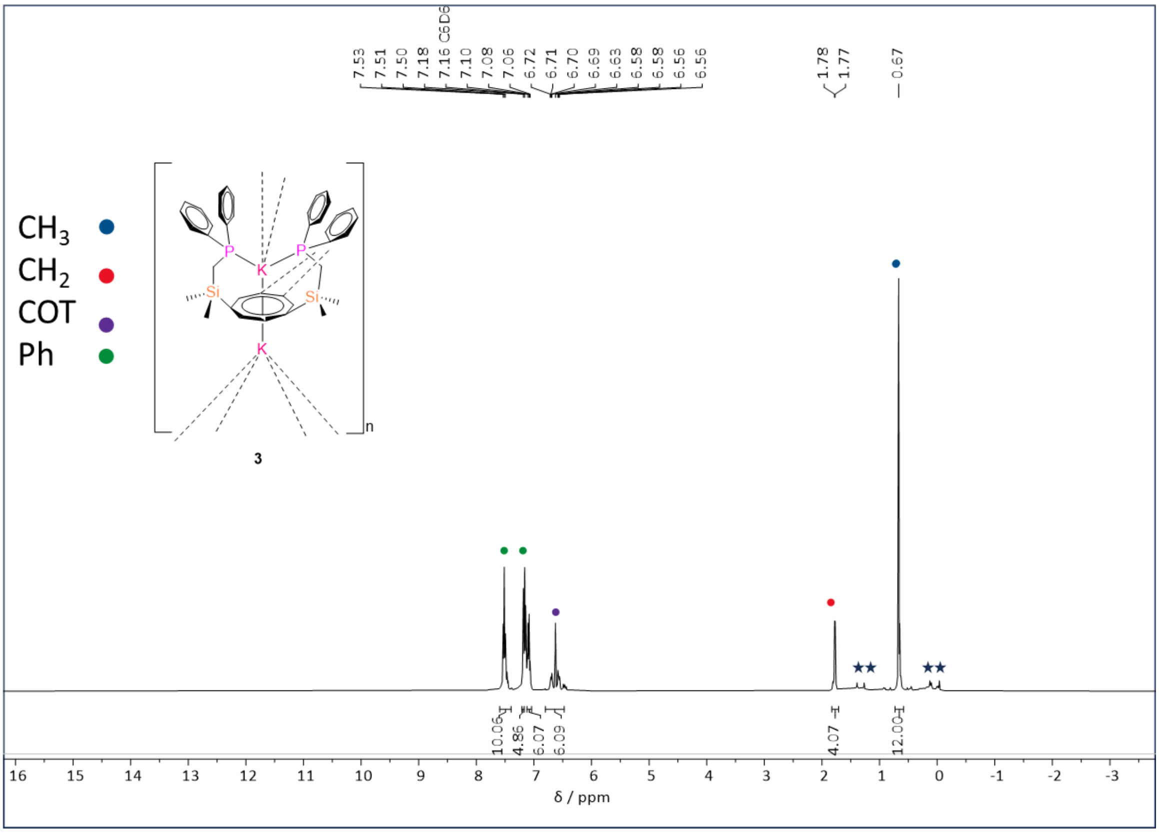

Figure S8: ^1H NMR spectrum of **3** in C_6D_6 at room temperature. (★) unidentified impurities.

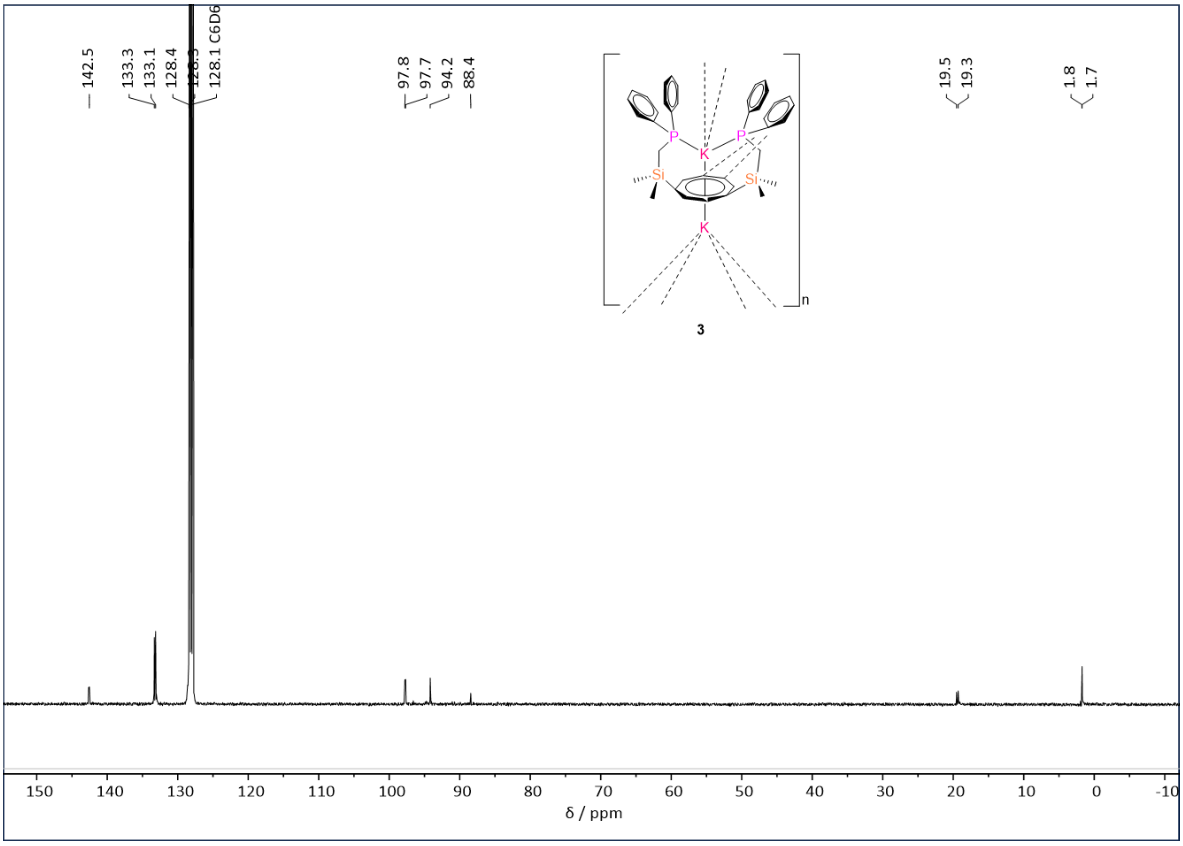

Figure S9: $^{13}C\{^{1}H\}$ NMR spectrum of **3** in C_6D_6 at room temperature.

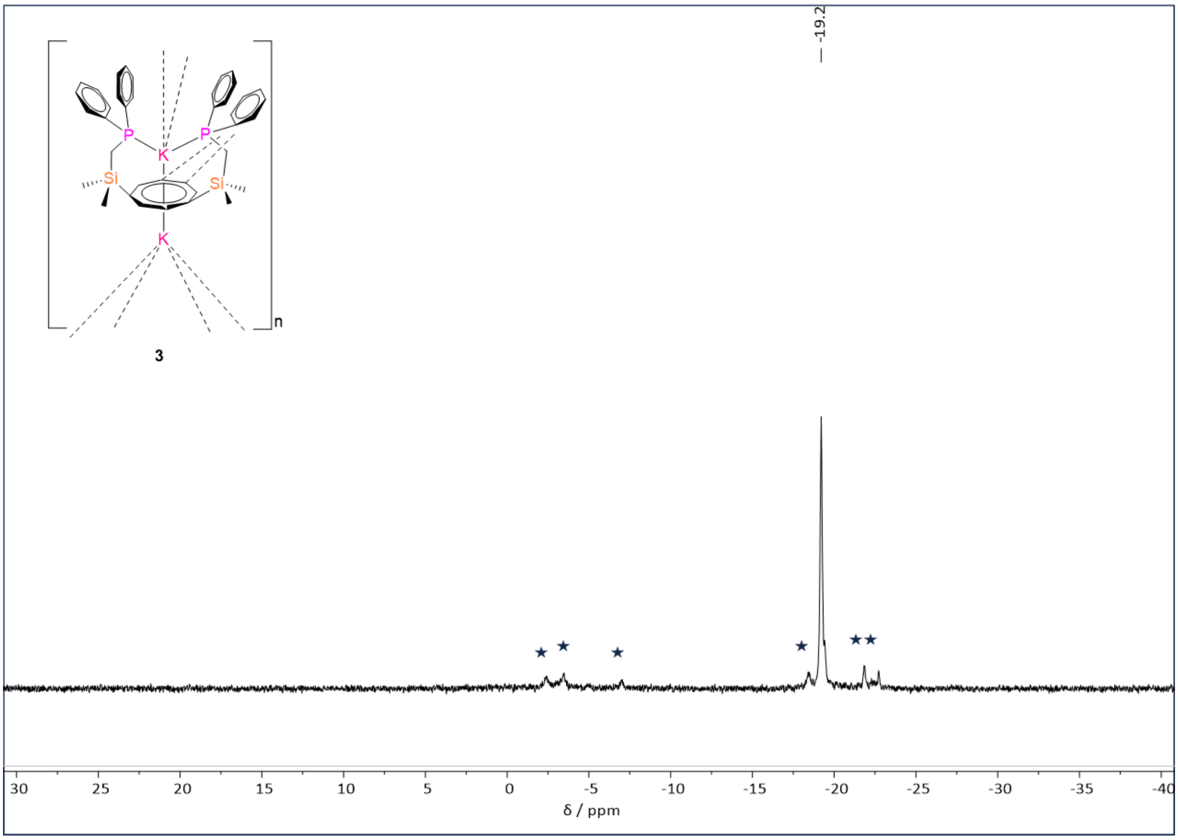

Figure S10: $^{31}P\{^{1}H\}$ NMR spectrum of **3** in C_6D_6 at room temperature. (★) unidentified impurities.

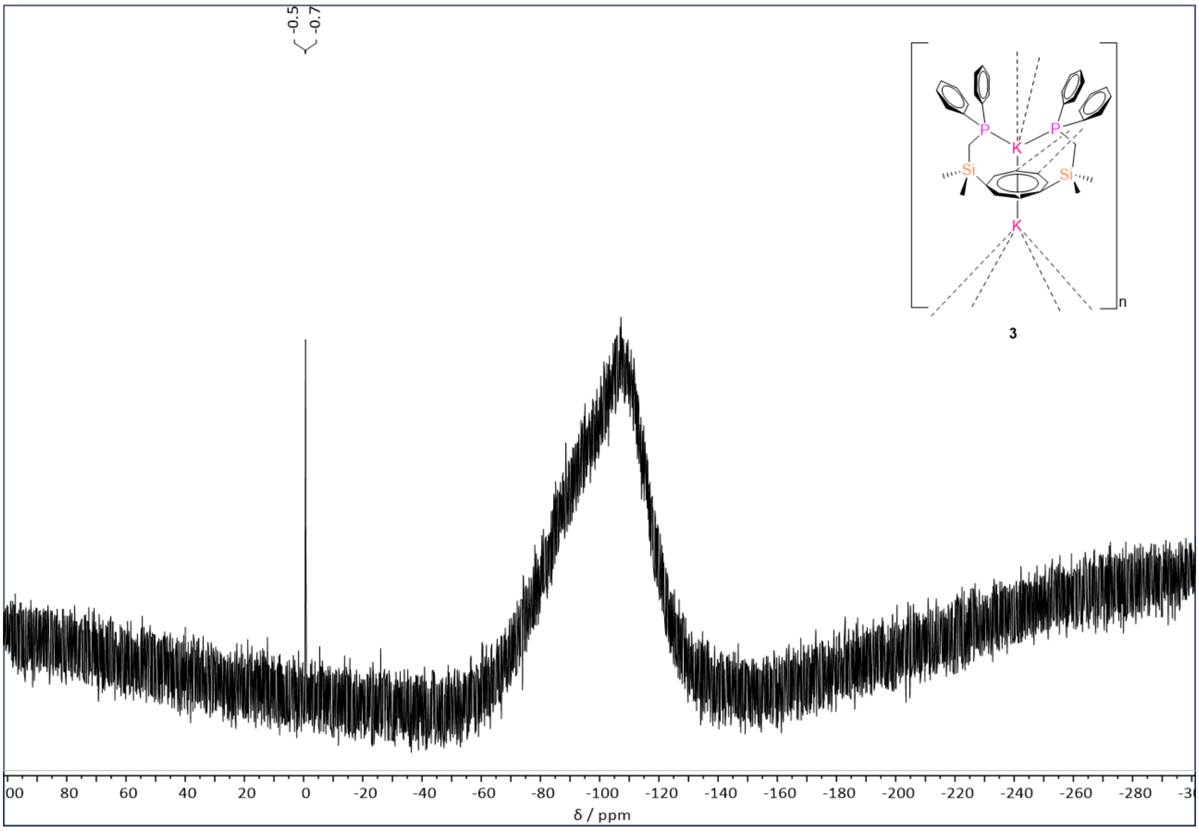

Figure S11: $^{29}Si\{^{1}H\}$ NMR spectrum of **3** in C_6D_6 at room temperature.

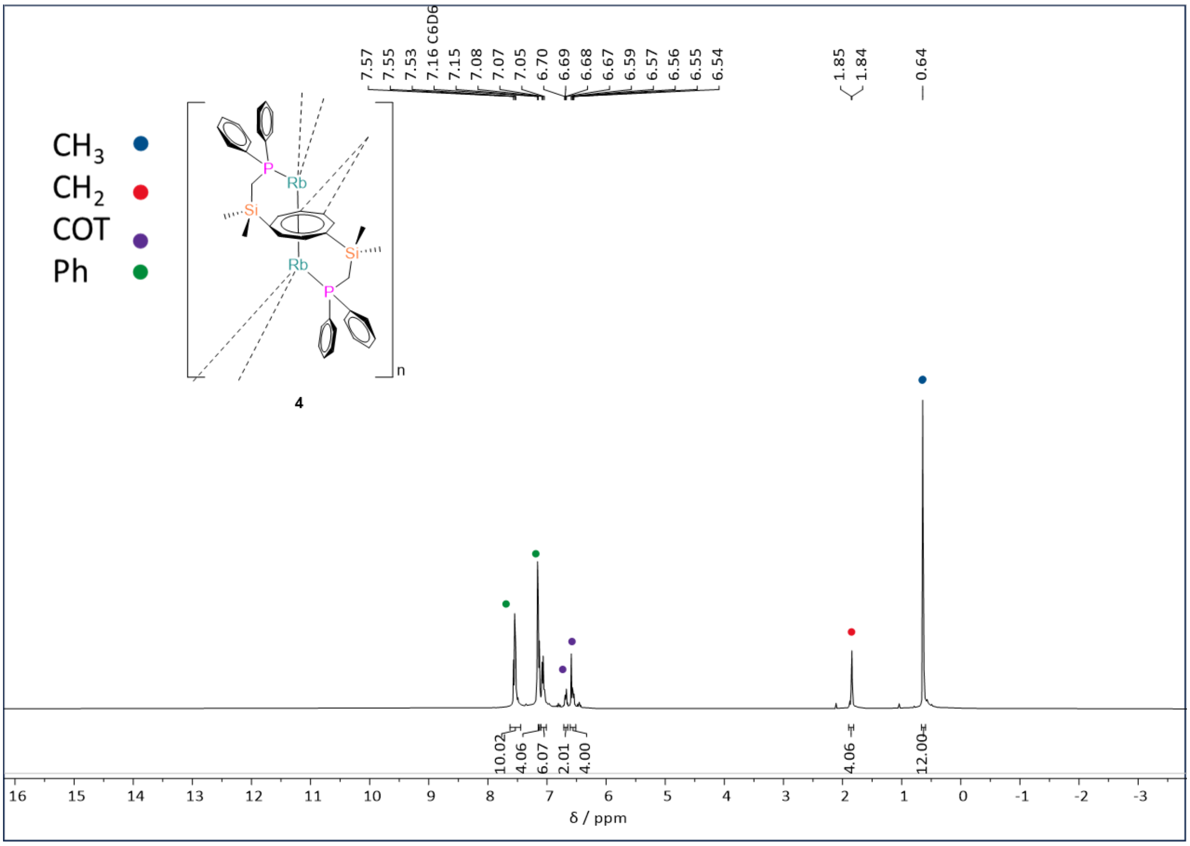

Figure S12: ^{1}H-NMR spectrum of **4** in C_6D_6 at room temperature.

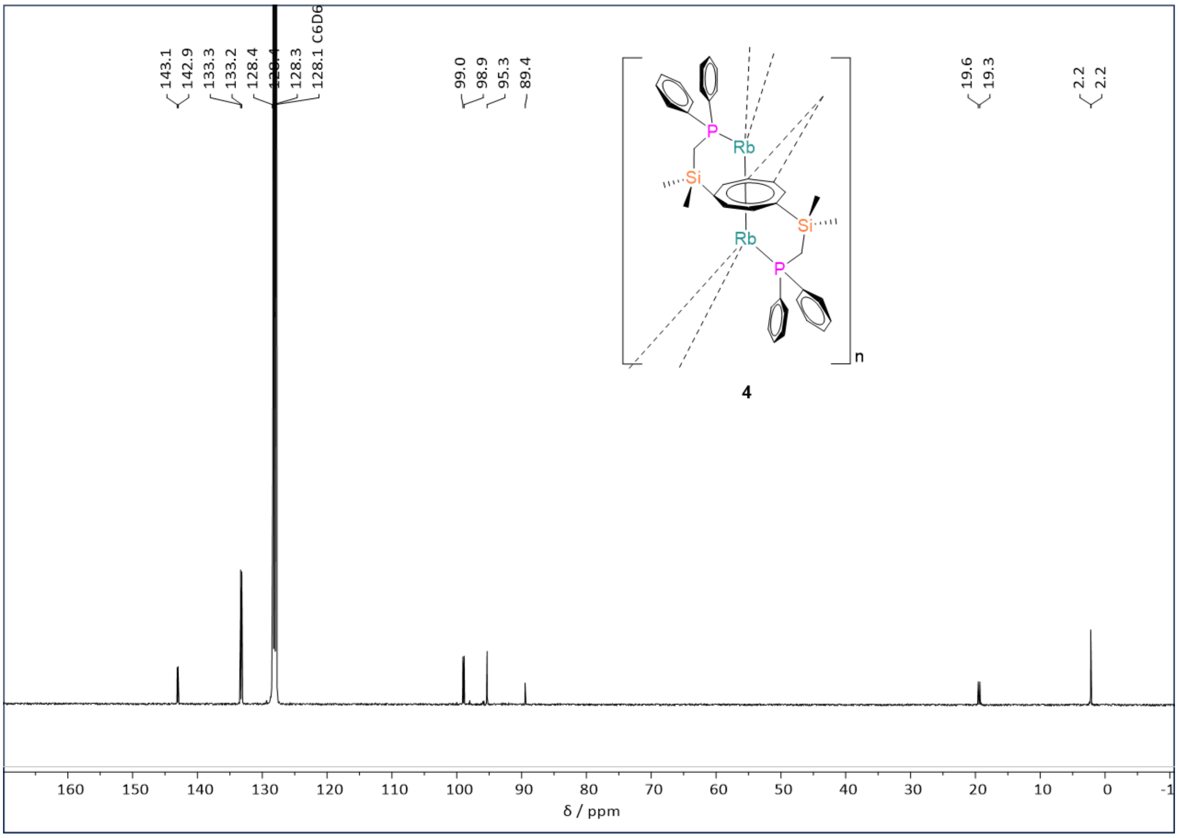

Figure S13: $^{13}C\{^1H\}$ NMR spectrum of **4** in C_6D_6 at room temperature.

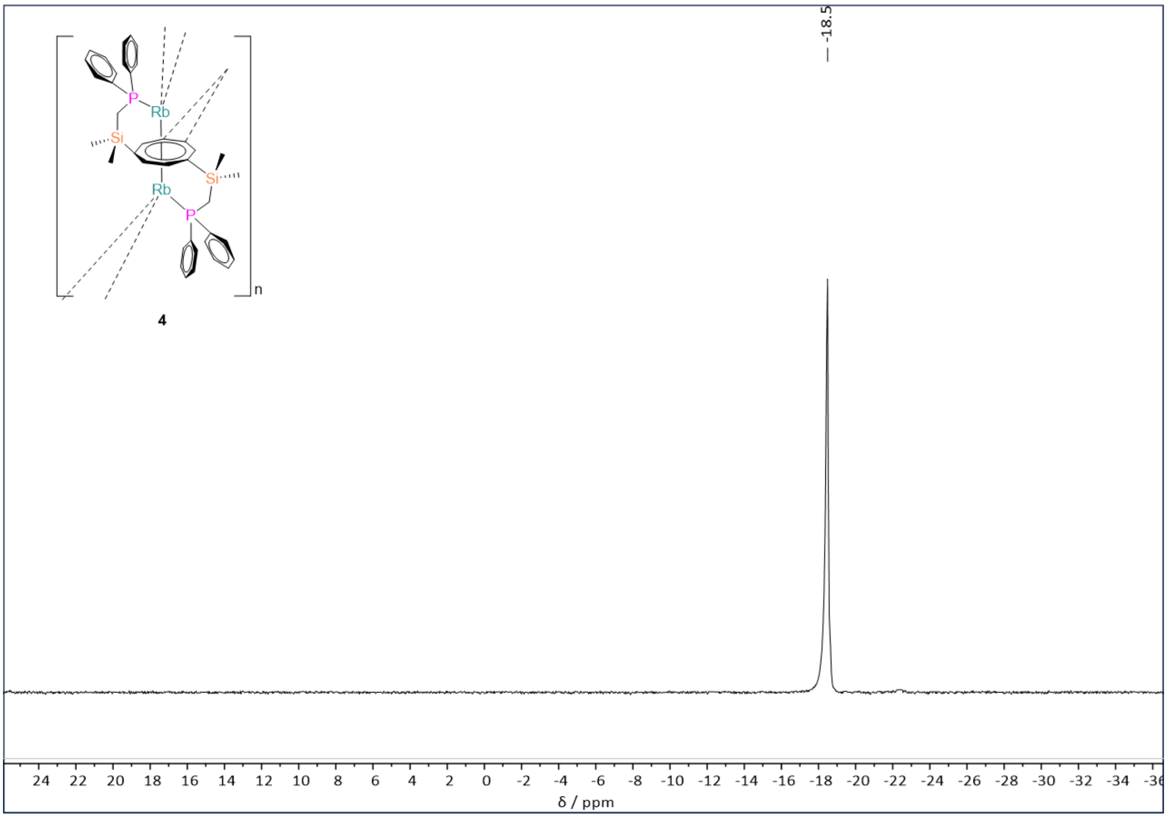

Figure S14: $^{31}P\{^1H\}$ NMR spectrum of **4** in C_6D_6 at room temperature.

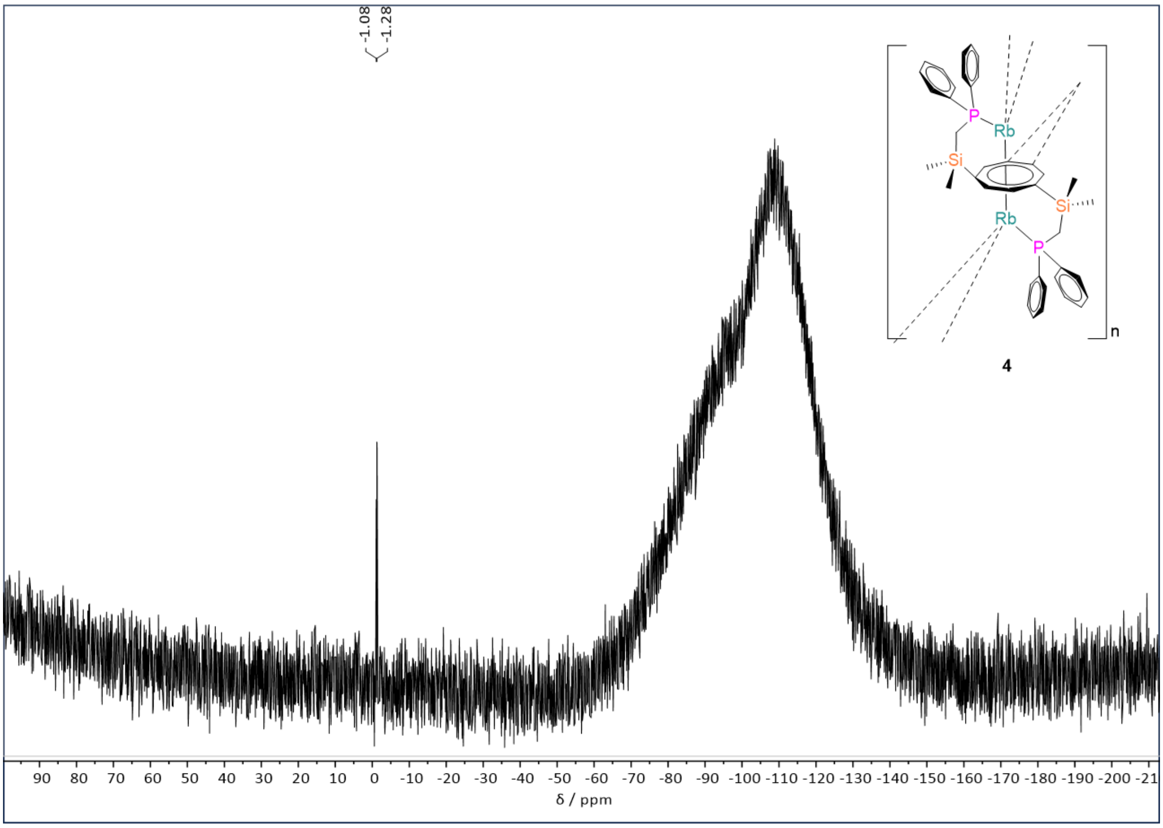

Figure S15: $^{29}Si\{^1H\}$ NMR spectrum of **4** in C_6D_6 at room temperature.

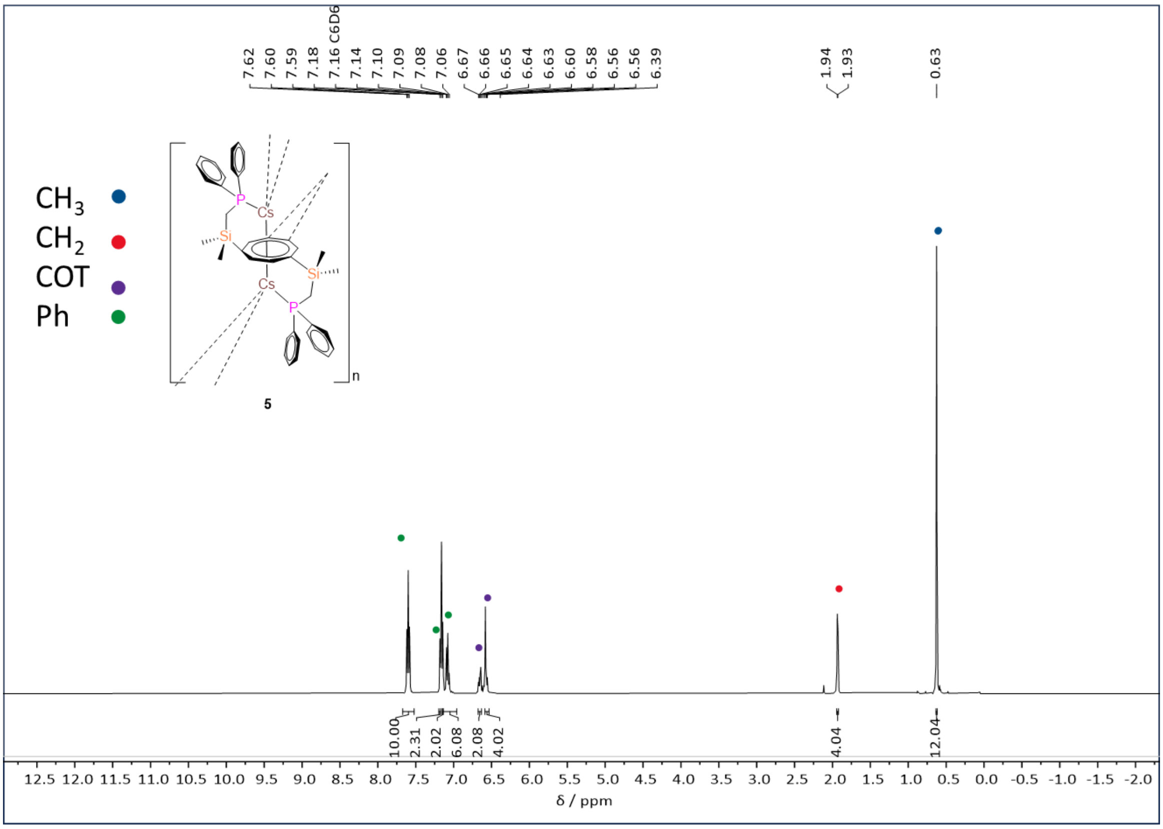

Figure S16: ^1H-NMR spectrum of **5** in C_6D_6 at room temperature.

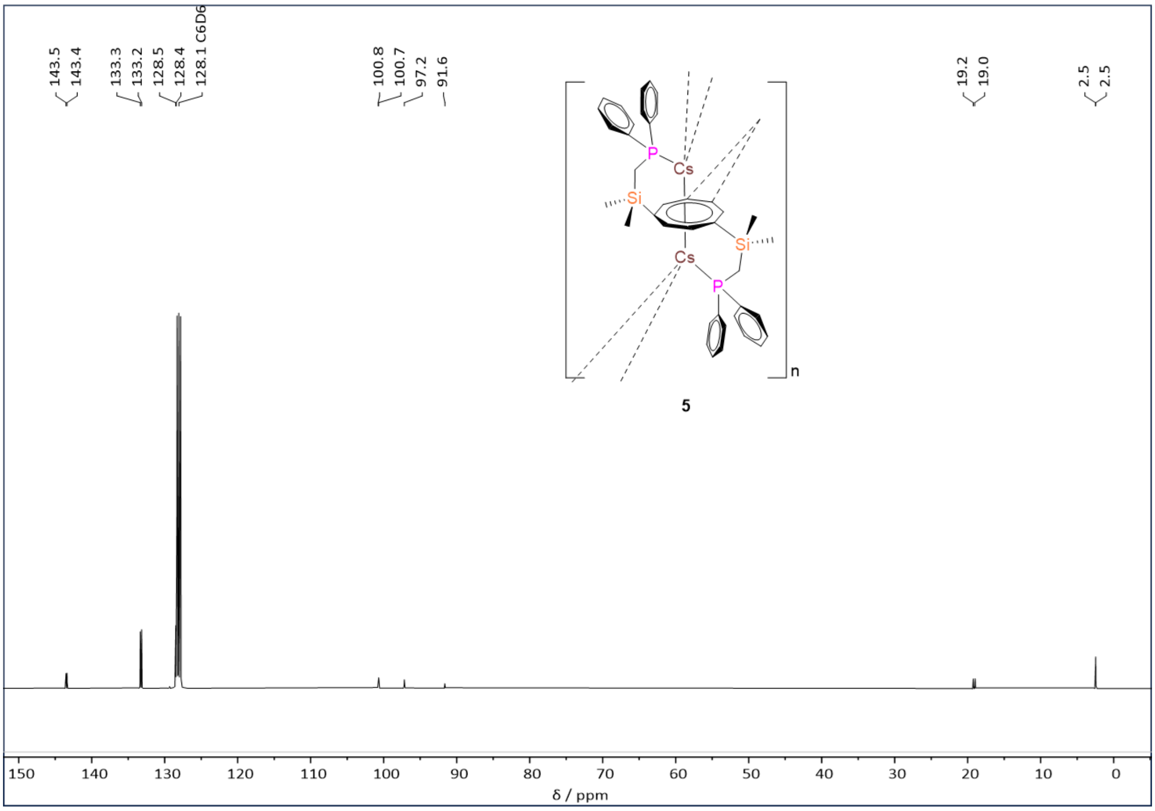

Figure S17: $^{13}C\{^1H\}$ NMR spectrum of **5** in C_6D_6 at room temperature.

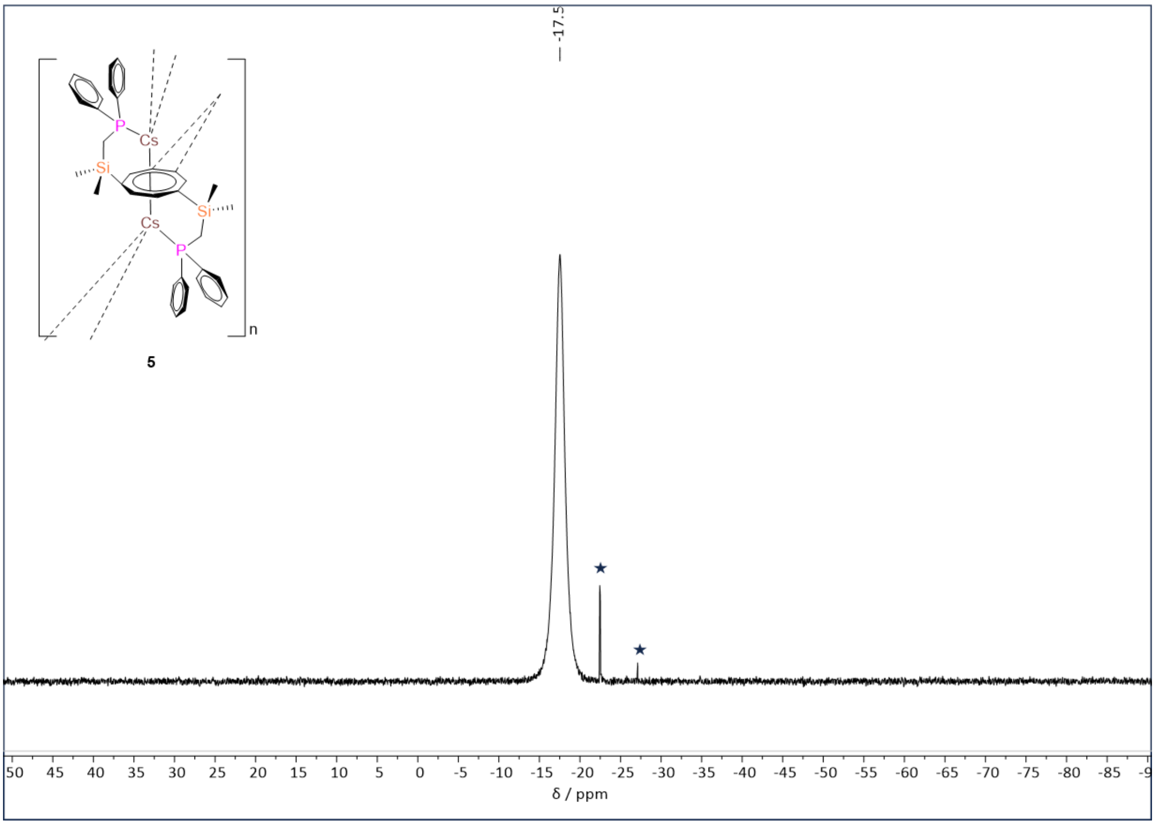

Figure S18: $^{31}P\{^1H\}$ NMR spectrum of **5** in C_6D_6 at room temperature. (★) unidentified impurities.

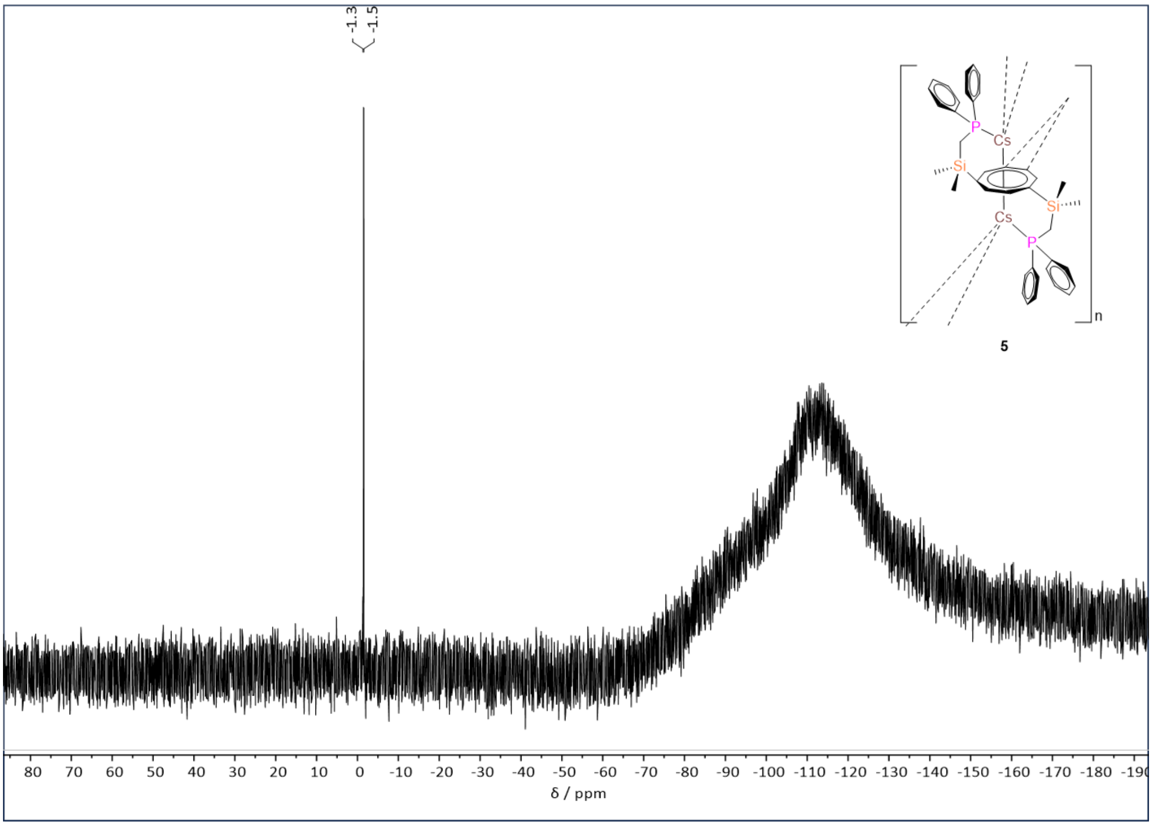

Figure S19: $^{29}Si\{^{1}H\}$ NMR spectrum of **5** in C_6D_6 at room temperature. (★) unidentified impurities.

4. IR Spectra

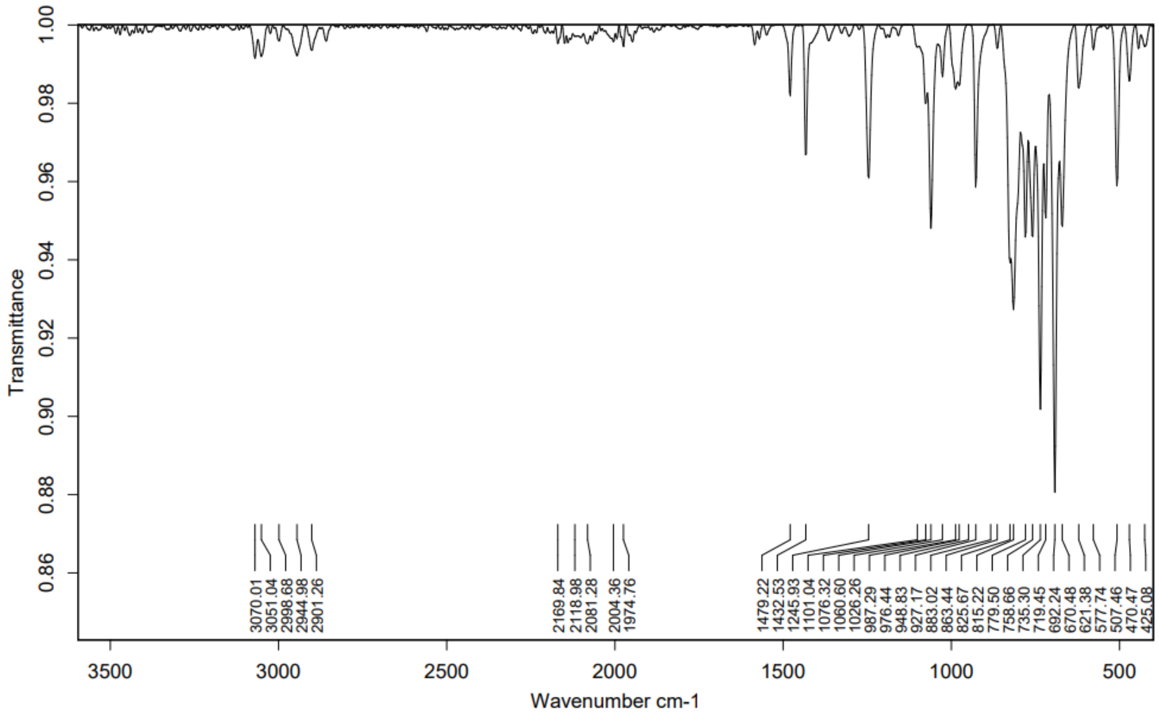

Fig S20: IR Spectrum of complex **2**

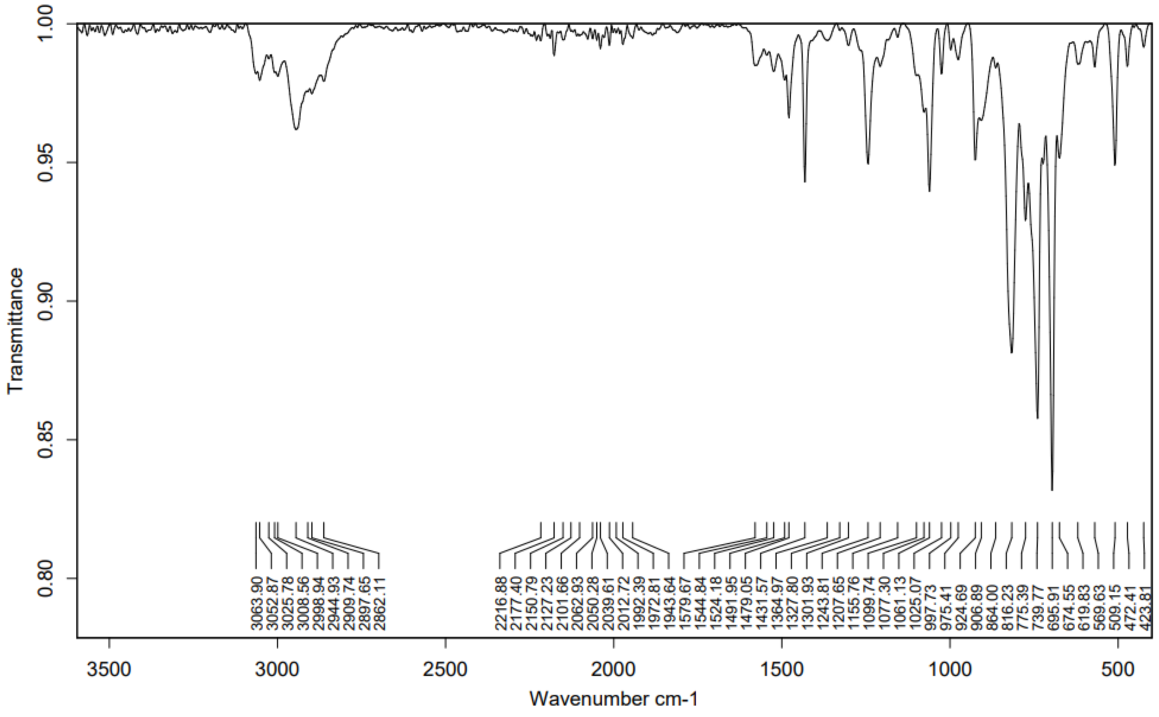

Fig S21: IR Spectrum of complex **3**

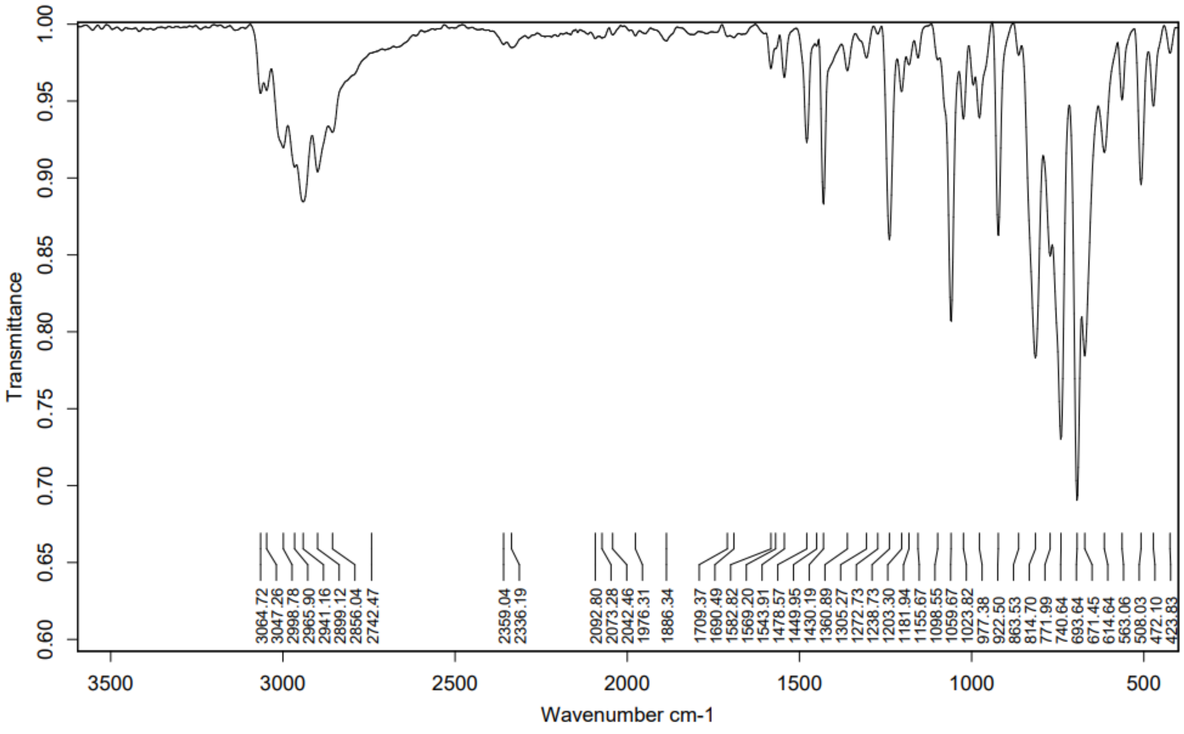

Fig S22: IR Spectrum of complex **4**

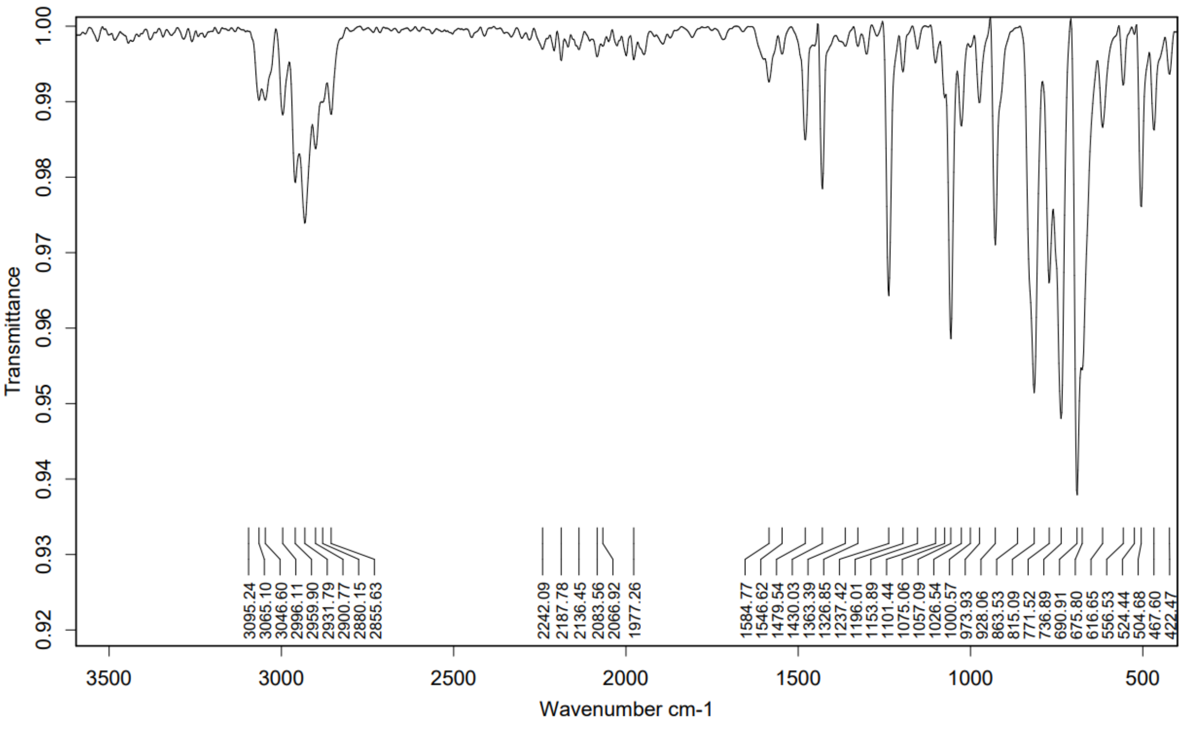

Fig S23: IR Spectrum of complex **5**

5. X-ray crystallography studies

Suitable crystals for the X-ray analysis of all compounds were obtained as described above. A suitable crystal was covered in mineral oil (Aldrich) and mounted on a glass fiber. The crystal was transferred directly to the cold stream of a STOE StadiVari (100 K or 150 K) diffractometer. All structures were solved by using the program SHELXS/T [3, 4] and Olex2.[5] The remaining non-hydrogen atoms were located from successive difference Fourier map calculations. The refinements were carried out by using full-matrix least-squares techniques on F^2 by using the program SHELXL.[3,4] The H-atoms were introduced into the geometrically calculated positions (SHELXL procedures) unless otherwise stated and refined riding on the corresponding parent atoms. In each case, the locations of the largest peaks in the final difference Fourier map calculations, as well as the magnitude of the residual electron densities, were of no chemical significance. Summary of the crystal data, data collection and refinement for all compounds are given in Table S1.

Crystallographic data for the structures reported in this paper have been deposited with the Cambridge Crystallographic Data Centre as a supplementary publication no. 2457212-2457215 Copies of the data can be obtained free of charge on application to CCDC, 12 Union Road, Cambridge CB21EZ, UK (fax: (+(44)1223-336-033; email: deposit@ccdc.cam.ac.uk)

Table S1: Crystal data and structure refinement for **3-5.**

**Compounds**	**3**	**4**	**4'**	**5**
**Formula**	$C_{45}H_{50}Si_2P_2K_2$	$C_{38}H_{42}P_2Rb_2Si_2$	$C_{47}H_{51}P_2Rb_2Si_2$	$C_{38}H_{42}Cs_2P_2Si_2$
**Formula weight**	787.17	787.77	904.93	882.65
***T*/K**	100	100	100	100
**Crystal system**	Triclinic	Monoclinic	Monoclinic	Monoclinic
**Space group**	$P\bar{1}$	*C*2/*c*	$P2_1/n$	*C*2/*c*
***a*/Å**	11.884(2)	35.288(2)	13.6521(7)	35.3542(17)
***b*/Å**	12.294(3)	9.6423(4)	18.0420(8)	9.7083(3)
***c*/Å**	14.912(3)	11.3809(7)	18.9255(10)	11.6519(6)
***α*/°**	89.90(3)			
***β*/°**	88.58(3)	102.465(5)	107.416(4)	103.072(4)
***γ*/°**	79.17(3)			
**V/Å^3**	2139.3(8)	3781.2(4)	4447.9(4)	3895.6(3)
**Z**	2	4	4	4
**ρ_{calc} g/cm^{-3}**	1.222	1.384	1.351	1.505
**μ/mm^{-1}**	0.382	2.761	2.357	2.040
**F(000)**	832.0	1608.0	1860.0	1752.0
**Crystal size/mm^3**	0.211 × 0.133 × 0.076	0.079 × 0.053 × 0.025	0.146 × 0.1 × 0.025	0.382 × 0.253 × 0.094

**Radiation**	Mo Kα (λ = 0.71073)	Mo Kα (λ = 0.71073)	Mo Kα (λ = 0.71073)	Mo Kα (λ = 0.71073)
**2Θ range for data collection/°**	4.334 to 51.996	4.386 to 51.994	3.856 to 50.498	4.36 to 52
**Index ranges**	$-14 \le h \le 14$, $-15 \le k \le 15$, $-18 \le l \le 18$	$-43 \le h \le 43$, $-11 \le k \le 11$, $-14 \le l \le 14$	$-16 \le h \le 15$, $0 \le k \le 21$, $0 \le l \le 22$	$-42 \le h \le 43$, $-11 \le k \le 11$, $-14 \le l \le 13$
**Reflections collected**	23410	19240	7999	9609
**Independent reflections**	8371 [R_{int} = 0.0436, R_{sigma} = 0.0472]	3713 [R_{int} = 0.0883, R_{sigma} = 0.0835]	7999 [R_{int} = 0.0883, R_{sigma} = 0.1088]	3810 [R_{int} = 0.0210, R_{sigma} = 0.0227]
**Data/restraints/parameters**	8371/0/465	3713/0/201	7999/0/483	3810/0/201
**GooF**	0.999	1.063	1.023	1.075
**Final R indexes [I>=2σ (I)]**	R_1 = 0.0493, wR_2 = 0.1269	R_1 = 0.0520, wR_2 = 0.0875	R_1 = 0.0752, wR_2 = 0.1748	R_1 = 0.0283, wR_2 = 0.0769
**Final R indexes [all data]**	R_1 = 0.0673, wR_2 = 0.1382	R_1 = 0.1064, wR_2 = 0.1076	R_1 = 0.1361, wR_2 = 0.2168	R_1 = 0.0314, wR_2 = 0.0779
**Largest diff. peak/hole / e Å^{-3}**	0.68/-0.55	0.50/-0.70	1.24/-1.02	0.73/-1.28

Crystal Structures of 2

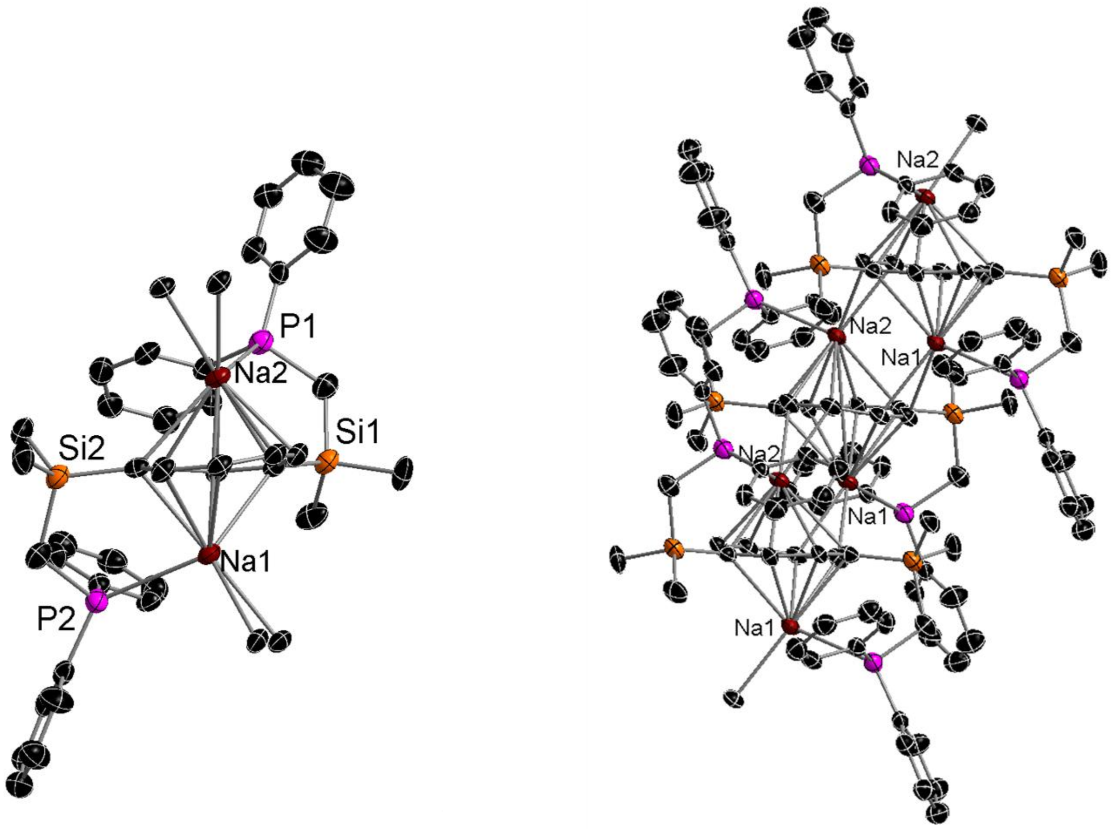

Figure S24: Molecular structure of complex **2** in the solid state. Hydrogen atoms and non-coordinating solvents are removed for clarity. Thermal ellipsoids are drawn at 50 % probability.

Crystal Structure of 3

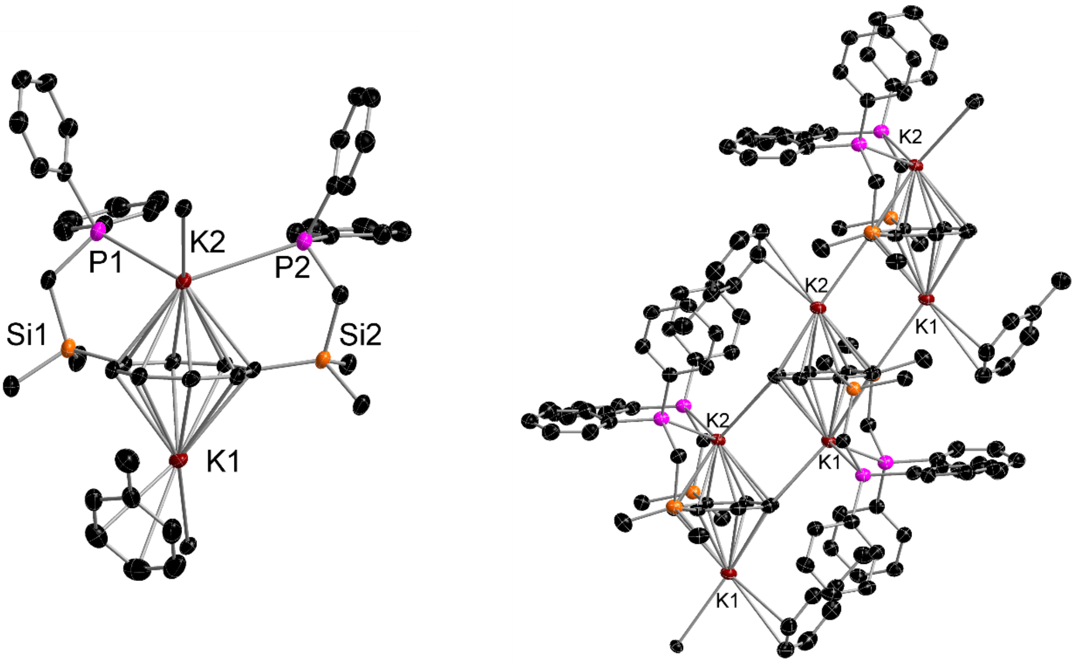

Figure S25: Molecular structure of complex **3** in the solid state. Hydrogen atoms and non-coordinated solvents were omitted for clarity. Thermal ellipsoids are drawn at 50 % probability. Selected bond distances (Å) and angles (°): K1-C_{cot} 2.895(2)-3.048(2), K2-C_{cot} 2.923(2)-3.014(2), K1-K2 4.692, C3-Si1 1.851(2) C6-Si2 1.854(2), C1-K11 3.134(2), C8-K11 3.208(8), K2-P1 3.429(14), K2-P2 3.388(13), K1-$C_{toluene}$ 2.392(3)-3.509(3); and P1-C-Si1 115.95(13), P2-C-Si2 115.22(12), C1-K1-C41 28.60(6) C1-K1-C42 88.65(8), C1-K1-C43 107.30(7).

Crystal Structure of 4

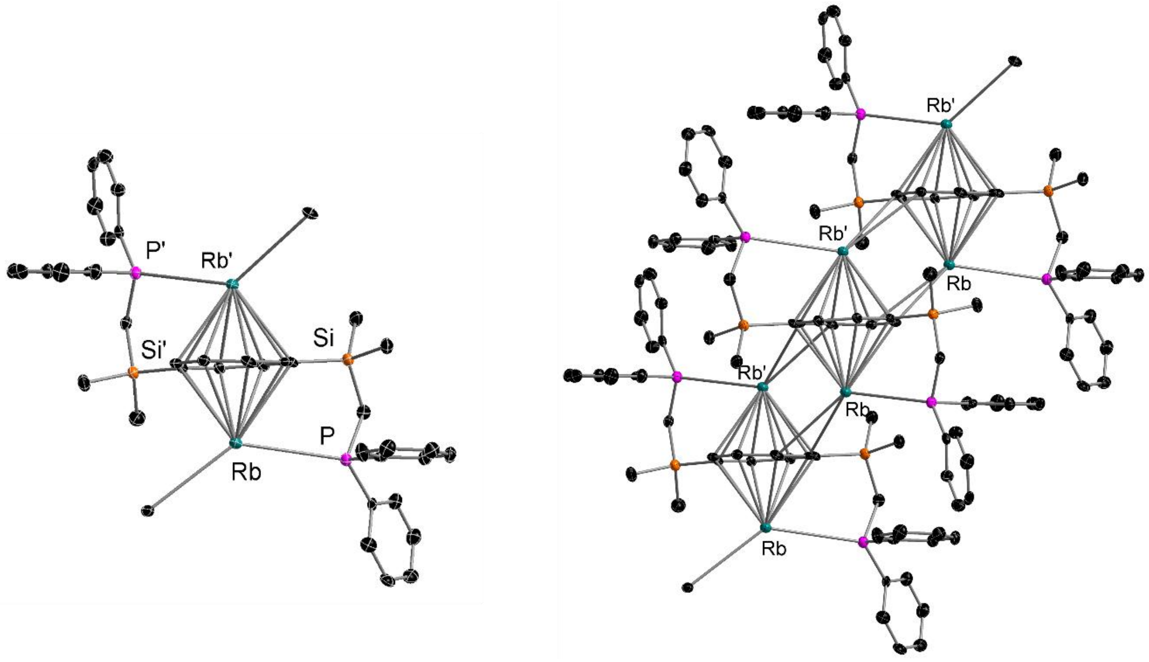

Figure S26 Molecular structure of complex **4** in the solid state. Hydrogen atoms and non-coordinated solvents were omitted for clarity. Thermal ellipsoids are drawn at 50 % probability. Selected bond distances (Å) and angles (°): Rb-C_{cot} 3.148(5)-3.073(5), Rb-Rb' 4.4937(10), C3-Si 1.851(5) C6-Si 1.851(5), C1-Rb 3.599(5), C2-Rb 3.451(5), Rb-P 3.638(14); and P1-C-Si1 113.3(2).

Crystal Structure of 4'

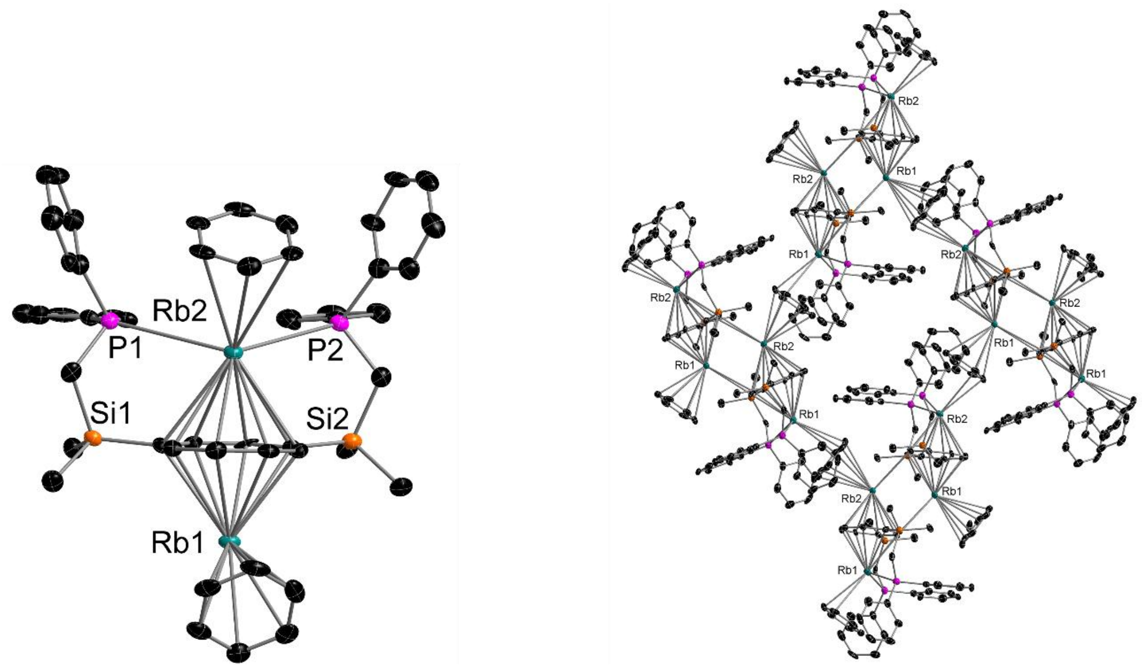

Figure S27: Molecular structure of complex **4’** in the solid state. Hydrogen atoms and non-coordinated solvents were omitted for clarity. Thermal ellipsoids are drawn at 50% probability. Selected bond distances (Å) and angles (°): Rb1-C$_{cot}$ 3.050(8) Å-3.174(8), Rb2- C$_{cot}$ 3.050(8) Å-3.174(8), Rb1-Rb2 4.4937(10), C4-Si1 1.842(9), C6-Si2 1.848(9), Rb1-P1 3.496(2), Rb2-P2 3.490(2), C27-Rb1 3.456(8), C28-Rb1 3.512(9), C29-Rb1 3.512(9), n^6C$_{benzene}$-Rb1 3.442(8)-3.473(9); and P1-C-Si1 113.9(5), P2-C-Si2 115.0(5).

Crystal Structure of 5

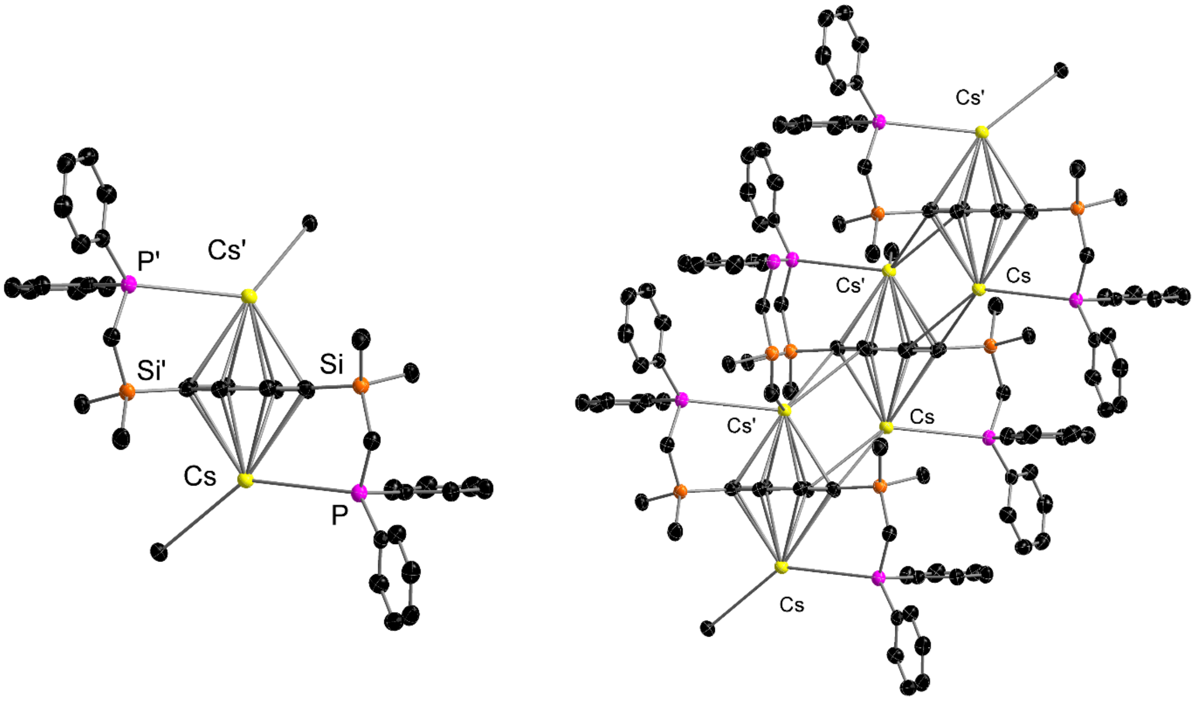

Figure S28: Molecular structure of complex **5** in the solid state. Hydrogen atoms and non-coordinated solvents were omitted for clarity. Thermal ellipsoids are drawn at 50% probability. Selected bond distances (Å) and angles (°): Cs-C_{cot} 3.169(5)-3.296(3), Cs-Cs' 4.5697(3), C3-Si 1.861(3), C6-Si 1.880(3), Cs-P 3.742(7), C1-Cs 3.639(2)-(3.567(2); and P-C-Si 113.80(14).

6. Photoluminescence measurements

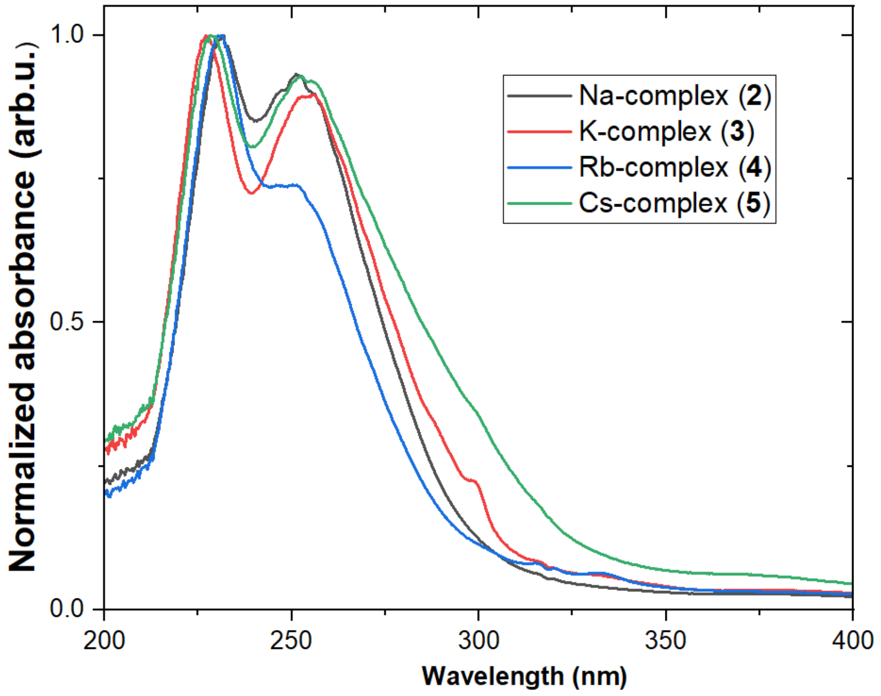

Figure S29: Normalized absorption spectra of complexes **2**, **3**, **4** and **5** in toluene at ambient temperature.

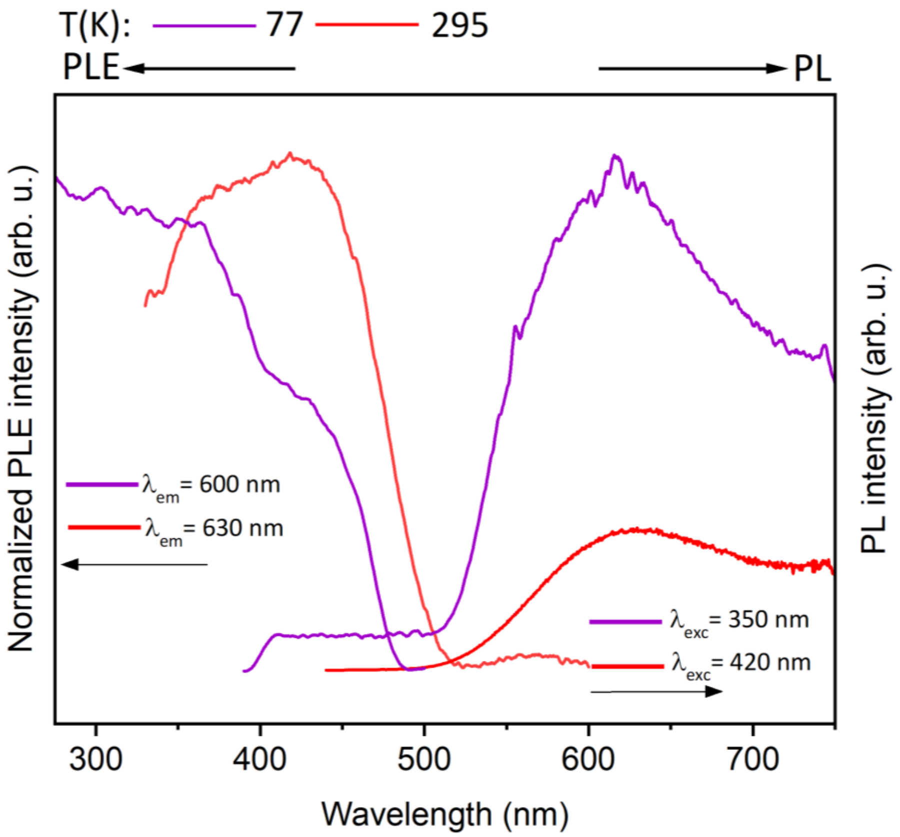

Figure S30: Photoluminescence excitation (PLE) and emission (PL) spectra of complex **4** in the solid state at 77 K and room temperature. PLE and PL spectra were recorded at the indicated emission and excitation wavelengths (λ_{em} and λ_{exc}, respectively).

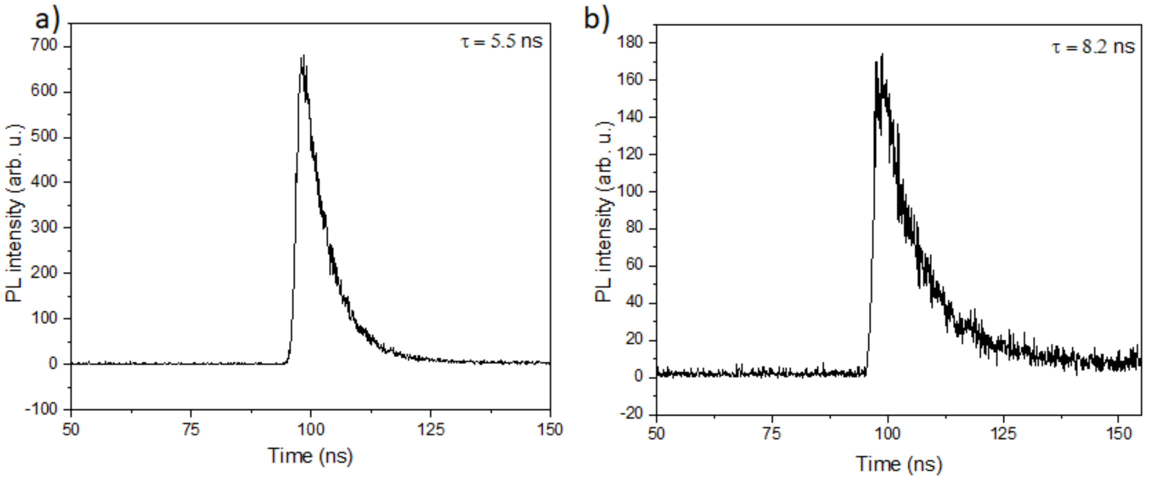

Figure S31: Kinetic traces of complex **2** in the solid state at a) room temperature and b) 77 K.

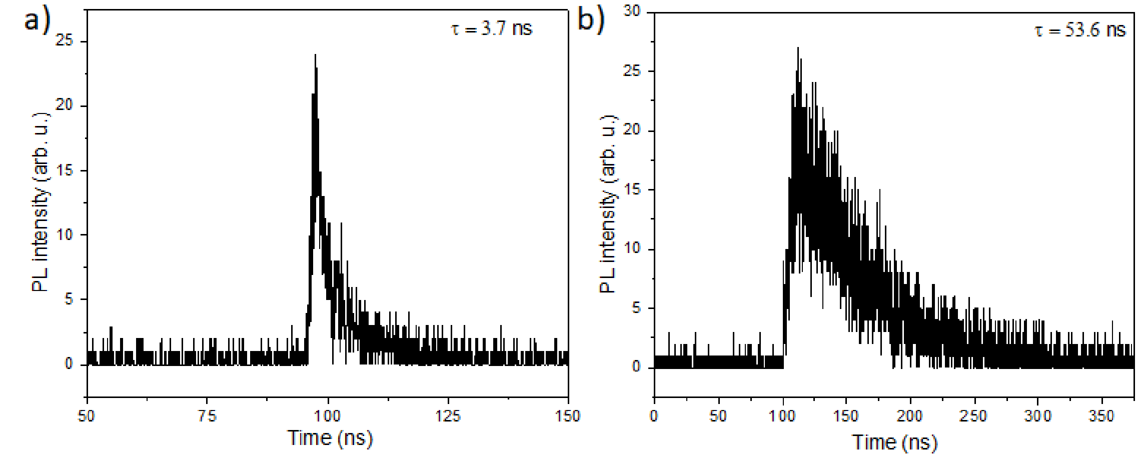

Figure S32: Kinetic traces of complex **5** in the solid state at a) room temperature and b) 77 K.

7. References

[1] H. H. Sønsteby, K. Weibye, J. E. Bratvold, O. Nilsen, *Dalton Trans.* **2017**, *46*, 16139-16144.

[2] W. Wei, B. Yu, F. Alam, Y. Huang, S. Cheng, T. Jiang, *Transit. Met. Chem.* **2019**, *44*, 125-133.

[3] G. M. Sheldrick, *Acta Cryst. A* **2008**, *64*, 112-122.

[4] G. M. Sheldrick, *Acta Cryst. C* **2015**, *71*, 3-8.

[5] O. Dolomanov, L. Bourhis, R. Gildea, J. Howard, H. Puschmann, *J. Appl. Cryst* **2009**, *42*, 339-341.
